# The 1991 International Symposium on Laboratory Automation and Robotics

**DOI:** 10.1155/S146392469100041X

**Published:** 1991


Journal of Automatic Chemistry, Vol. 13, No. 6 (November–December 1991), pp. 243–266

### The 1991 International Symposium on Laboratory Automation and Robotics

*Nearly 380 participants gathered in Boston for the ninth meeting held from 20 to 23 October 1991. They came from many parts of the United States and fourteen other countries. The format for the conference included plenary lectures, other oral presentations, poster papers and a visit to the facilities of the meeting hosts, Zymark Corporation in Hopkinton, Massachusetts. Following the keynote speakers on Monday morning, there were sessions on Pharmaceutical analysis and advanced technology. The former continued into the afternoon in parallel with session on Intelligent automation and microplates. Poster presentations completed the afternoon schedule. The programme on Tuesday followed a similar pattern. Morning sessions on Managing laboratory automation and Environmental ran in parallel. The latter continued on in the afternoon along with a session on Biology/biotechnology. Poster sessions again completed the afternoon programme. General papers were presented on Wednesday in parallel with a session on chromatography sample preparation.*

*The plenary programme featured two speakers from the pharmaceutical industry; David B. Weinstein of the Sandoz Research Institute and Bernd C. Schade of Miles Incorporated. Their topics were, respectively, 'Jumping into the 20th century before it's too late; is laboratory robotics still in its infancy?, and 'Quality control in the year 2000'. It is interesting that while pharmaceutical firms early on recognised the contribution robotics could make to laboratory automation, their focus is on the future.*

*Five of the six speakers in the session on Managing laboratory automation were also from the pharmaceutical industry. A theme that ran through the presentations from the pharmaceutical industry is the need for support of senior management in the development of robotic facilities and the fact that this commitment must be for the long haul. Of course, the bottom line is better quality products and increased productivity.*

*Another feature of the meeting is the presentation of the Pioneers in Laboratory Robotics Awards. The recipients for this year were:*

Timothy Lincoln (Xerox Corporation)
Mark Arnold (Bristol-Meyers Squibb)
Frank Dias (WMI Environmental Laboratories Inc)
Lisa Martin (ICI Pharmaceuticals)
Richard Young (RW Johnson Pharmaceutical Research Institute)
Katherine Ku (Schering Plough)
Mike Metzger (The Goodyear Tire & Rubber Company)
Stephen Conder (Bristol-Myers Squibb)
Kevin J. Halloran (Carter-Wallace Inc)

*The abstracts of the full programme are included in this issue of* Journal of Automatic Chemistry *by kind permission of the Zymark Corporation – and the papers will be available in early 1992 as 'Advances in Laboratory Automation and Robotics', Vol. VIII.*

*This meeting is well worth attending. The tenth symposium will be held from 25 to 28 October 1992 and information is available from Sharon Correia, Zymark Corporation, Zymark Center, Hopkinton, Massachusetts 01748, USA. Tel.: 508 435 9500. Fax: 508 435 3439.*

DAVID J. CURRAN
*American Editor*

### *Abstracts from the 1991 International Symposium on Laboratory Automation and Robotics*

### KEYNOTES

#### Jumping into the 20th century before it's too late: is laboratory robotics still in its infancy?

*David B. Weinstein and Dennis S. France (Sandoz Research Institute, East Hanover, NJ 07936, USA)*

Successful management of laboratory robotic automation programs in the area of research and drug discovery in the pharmaceutical industry may perhaps be best compared to a chef preparing the perfect sauce. All the ingredients must be available at the same time and be of highest quality for the right price. However, if components are not added in the right quantities, and in the proper order, no amount of whipping together by the product champion will create the best product. In the past, managerial scepticism surrounding useful implementation of cost-effective, high throughput robotic systems often placed these 'modern toys' at low priorities for research development laboratories. Management now recognizes the unique contributions of robotics in the research environment. Although the scientific director must still play the role of product champion, new questions are being proposed and new commitments are being made to bring the potential of robotic automation to every laboratory where repetitive functions can benefit from new applications. Research laboratory directors have become both the key ingredient, as well as the rate-limiting determinant in the development of new applications. Having fulfilled the promise of robotic automation to release talented personnel, the challenge now is for the 'end users', the bench scientists, to be provided with opportunities to critically invest time and effort required for future applications and new career functions.

### Quality control in the Year 2000

*Bernd C. Schade (Miles, Inc., Consumer Healthcare Division, Elkhart, IN 46515, USA)*

'Just-In-Time' production is a prerequisite for a copy to meet the challenges of competition. Manufacturing cycles have been so successfully optimized, that release time has become a significant factor. A vision for a major QC contribution to profitability in this decade seems to be the Just-In-Time-Release. Benefits will exceed cost savings for lower inventory, because problems are detected as they occur. To meet this goal, the requirements for automated instruments will change significantly. Quality, reliability, durability, and ruggedness of automated systems will be crucial factors for success.

---

### PHARMACEUTICAL ANALYSIS

### Better chemistry through robotics: an automated system for process optimization

*Susan D. Boettger (Bristol-Myers Squibb Company, Syracuse, NY, USA)*

Rapid development of an economical process for manufacture of the active ingredient plays a key role in the successful launch of a new pharmaceutical. The evolution from a multistep laboratory synthesis of milligram amounts to the streamlined manufacture of multikilogram quantities is typically a slow and labour-intensive process. The Bristol-Meyers Squibb Process Optimization Robotic System was designed to shorten this process by increasing the quantity and quality of reactions investigated. In addition, automated reaction setup and monitoring frees the development chemist for more creative activities.

This author described the three-phase development of the Process Optimization Robotic System. In Phase 1, automation of a 2 h, room temperature reaction was achieved via hardware and software modifications of a custom Zymark PyTechnology system. The second phase involved installation of a low temperature cooling system and control of reaction temperatures from −60 °C to 125 °C via EasyLab programming. Phase 3 comprised optimization of a palladium-catalysed reaction by varying concentration, temperature and the amount and type of catalyst and additives. The ability to quantitatively assay multiple reactions during overnight runs resulted in reduction of the reaction time from 20 to 2 h and rapid identification of optimal reagents and temperature.

### Robot automation of routine assays in the stability testing of solid dosage forms

*Steven Conder (Bristol-Myers Squibb, New Brunswick, NJ, USA)*

The increasing demands of stability testing of dosage forms during drug development have created an immense sample load for routine assays such as *in vitro* dissolution testing, composite and single tablet High Performance Liquid Chromatographic (HPLC) assays, and Karl Fischer moisture analysis. The productive capacity of the laboratory which uses manual or semi-automated methods is easily overwhelmed by the volume of samples generated by these studies. The need for fast, accurate, and flexible automation capable of solving the variety of analytical problems facing the laboratories involved with stability testing is necessary to speed drug development. The New Brunswick Analytical Research & Development of Bristol-Myers Squibb has designed a laboratory dedicated to robot automation of the routine time-consuming assays involved with stability testing. The laboratory was designed to contain up to nine robots. Currently six Zymark robots and a Source for Automation (SFA) tablet extraction workstation are operational. The Zymark system types include four dissolution testing robots, one HPLC assay robot and a dual-function robot for Karl Fischer moisture determinations and tablet assays by wet-homogenization. This paper discusses several examples of successful assays performed by these robots, including the problems of system validation and sample/data management. The laboratory was successful in completing over 20 000 tablet assays in its first two years of operation.

### Robotic moisture determination of solids in the pharmaceutical industry

*Edward G. Kanczewski, Marie Skrilec and Robert E. Daly (Parke-Davis Pharmaceutical Research Division, Warner-Lambert Company, Morris Plains, NJ, USA)*

Accurate moisture determination of formulation excipients and active ingredients is a major concern in the pharmaceutical industry. Unassisted Karl Fischer Titrations are performed using a Zymark robotic system and appropriate Metrohm equipment. Samples having a range of moistures from approximately 0·5% to 10% are analysed in triplicate to insure precise and accurate results. User-friendly screens prompt the operator and allow the novice to use this system. Restricted access to Zymark software minimizes the curious from modifying system operating software.

### 'On-line' monitoring of a pharmaceutical tablet press using a customized laboratory workstation

*D. Jeff Roberts, Michael A. Shirley and Frederick K. Bangert (Bristol-Myers Products Division of Bristol-Myers Squibb Company, 9707 Chapel Hill Road, P.O. Box 300, Morrisville, NC 27560, USA)*

The method for determining Uniformity of Dosage (Content Uniformity) for solid dosage form pharmaceutical products, as defined in USP XXII, was automated using a customized Tablet Assay Workstation. The workstation performs all of the sample and glassware manipulation including HPLC analysis, cleaning and disposal. The system handles all sample transfers, as well as the addition of diluting solutions and internal standards. Sample analysis and quantitation is performed by an on-line HPLC injection and data collection system. The sample transfers and diluting solution additions are made with calibrated syringes, peristaltic pumps and automated pipetting hands, and the transfers are verified gravimetrically. In addition, this system has the ability to wash and dry the sample-processing containers for re-use by the system.

The goal is to incorporate the on-line sampling and physical testing (weight/thickness/hardness) capability of an automated tablet press with the capabilities of the Tablet Assay Workstation to generate real-time data for monitoring the performance of the tableting process. Ultimately, batch production and testing will be dropped in favour of continuous production and testing. The primary benefit of this system merger will be an overall increase in plant productivity through improvement of Quality Assurance throughput time, without compromising product integrity or process control, whilst meeting USP XXII Uniformity of Dosage Units requirements.

### Automated performance of particulate matter testing for parenteral products

*Thomas A. Barber, Christine Pavek-Hicks and John G. Williams (Baxter Healthcare Corporation, Round Lake, IL, USA)*

The enumeration of particulate matter in parenteral products provides a critical assessment of process cleanliness and product quality. The *United States Pharmacopeia* (*USP*) defines limits for allowable numbers of particles in both large volume parenteral (LVP) solutions and small volume injections (SVI). It also describes a test method for particulate matter based on light obsuration particle counters; this method is extensively applied by pharmaceutical manufacturers as a quality assurance and release assay.

The goal of the automation project described in this paper was the elimination of the manual component of the instrumental particulate matter test routinely performed on large numbers of samples. This was achieved by combining a Zymate robot with a HIAC particle counter. The performance criteria for the system include: (1) unattended operation; (2) fail-safe measures preventing incorrect unit identification; and (3), of extreme importance, logic which allows scrutiny of counts collected for artifacts before data is sent to a database. Due to the transfer of this work to a Zymate System, the technicians responsible for a test now spend only the time necessary to set the system up and load units to be tested. Additional benefits include improved precision of data and the ability to test outside of normal working hours.

### Automation of dissolution testing using robotics and network-based data management

*R. Foester, L. Nemec and J. Rosen (Schering-Plough, Kenilworth, NJ, USA)*

The requirements for dissolution testing have increased dramatically in recent years. At Schering-Plough Pharmaceutical Research, dissolution testing has been assigned to a specialized group which supports all solid dosage formulation research and development/improvement preceding product approval. Only through extensive automation and electronic data handling could adequate support and productivity be provided.

Dissolution testing involves the following four categories of laboratory work:

(1) Dissolution test set-up and clean-up.

(2) Dissolution run (basket, paddle) with sample collection.

(3) Quantitative analysis (UV, HPLC).

(4) Sample data management.

The majority of samples are tested semi-automatically on the equipment described below. The semi-automatic approach cannot handle the first task: test set-up (preparation of dissolution media, filling of vessels) and the final vessel clean-up. Integration of two Zymark dissolution robots into the system permits automatic vessel filling and clean-up for the four dissolution apparatuses serviced by robots.

Samplers and timers were designed for the semi-automatic sample aliquot collection. Trays holding sample containers are transferred from the samplers to the sippers attached to the spectrophotometer. An electronically controlled valve connected to house vacuum is used to sip aliquots through a flow-through cell on the spectrophotometer. No sample dilutions are necessary – the absorbance of aliquots is kept within quantitative limits by the selection of spectrophotometric flow cells with suitable optical path length.

Quantitative analysis of dissolution sample aliquots can be performed either on-line, during the actual dissolution runs (several commercial systems are available) or after aliquots have been collected in individual sample containers. For the semi-automatic mode we chose the latter approach since one spectrophotometer can easily run assays of samples collected on four or more dissolution apparatuses. The dissolution robots can operate either on-line or off-line, depending on the types of sample preparation and quantitation required (additional filtration, HPLC assays require off-line sample collection).

The data management portion of the system (custom-designed dissolution LIMS) has been instrumental in making highly productive and reliable data reporting possible. Samples are run using stored descriptors which are decoded by the software to select the appropriate assay method. Seven identically equipped IBM PC-based workstations are on-line and connected by a Local Area Network residing on a server equipped with a large storage capacity an automatic tape back-up assuring permanent and secure storage of all primary spectral and dissolution data.

The automation of dissolution testing at Schering-Plough required careful optimization of resources and a considerable amount of hardware and software design. The present approach is just one of many alternatives possible, all of them requiring extensive customization, since a viable, high-volume commercial system is not readily available.

### Automated HPLC analysis using a Zymark Robotic System

*K. K. Chow and Aurobindo Nair (Glaxochem, Ltd) and P. C. Thiak and Teoh Sek Hong (Microstate, Singapore)*

This paper presented the approach taken in automating a high volume, labour intensive operation. The application is in the quality control laboratory of Glaxochem (Pte) Ltd in Singapore. It involves the HPLC assay of the H2-antagonist, Ranitidine HCL, which is the active ingredient in the prescription drug, Zantac.

The manual HPLC assay is a tedious and time-consuming operation. This analytical routine has been automated by interfacing a Zymate II Robot, with the appropriate PySections, to a HPLC system. The robot handles all aspects of sample preparation and subsequent injection onto the HPLC system. This configuration has dramatically improved sample turnaround time and enhanced the accuracy and precision of the analytical results.

Aspects of the application programme and procedure undertaken in validating the complete system were discussed. Problems encountered during validation and the steps taken to overcome them were highlighted; and the utility of gravimetric laboratory techniques in improving accuracy and precision of analytical results was also explained.

---

### ADVANCED TECHNOLOGY

### Computer validation: how much is enough?

*James E. Curley (Pfizer, Inc., Groton, CT, USA)*

The author discussed the type of computer system that need validation from the managerial point of view of balancing costs and benefits. The requirements of those demanding validation must be taken into account in planning the experiments and documentation for validation. Approaches to developing test protocols, test cases, change control and documentation were described.

### New analytical technologies and the challenge to automation

*W. Jeffrey Hurst and Robert A. Martin, Jr. (Hershey Foods Technical Center, Hershey, PA, USA)*

Many discussions of laboratory robotics and automation concentrate on the use of robotics in sample preparation, but fail to discuss their integration into the overall analytical method. In recent years there have been rapid advances in several new analytical technologies. Two of the most intriguing developments have been Supercritical Fluid Extraction (SFE) and Capillary Electrophoresis (CE). Each of these technologies has its own set of unique problems. Additionally each has limited automation capabilities and therefore presents challenges to the development of next generation automated systems.

This presentation discussed the new technologies that are available and their possible integration into automated systems. Automation challenges for the 1990s were also discussed.

### Hands-free GC-Zymate to chromatography data system via Ethernet

*C. J. Deakin and M. J. Crook (BP Research Centre, Sunbury-on-Thames, UK)*

Many laboratory procedures can be automated to give significant improvements in accuracy, precision, speed of analysis and sample throughput. This automation invariably involves a variety of software controlled equipment which is rarely fully integrated into a completely automated laboratory.

The analysis of samples by GC Simulated Distillation involves extensive sample preparation, GC analysis, data collection and post analysis calculation. The complete analysis has been fully integrated through the development of in-house software to interface a Zymate PyTechnology automated sample preparation system with the VAX/VMS based VG DataSystem Chromatography data system (Multichrom). The robot controller PC has been connected to the site computer network via Pathworks for DOS software and the DECnet transport protocol. Using this approach to the transfer of sample parameters between devices has eliminated any manual re-entry or computational reformatting of the data. The VAX/VMS component of the software is installed as a DECnet object on the VAX node and is activated from the PC. Subsequent communication is via non-transparent task-to-task link between the two processors.

The advantages of this integrated approach were discussed, with special emphasis on the communications link between microprocessor based equipment from several vendors.

---

### INTELLIGENT AUTOMATION

### The analytical director project

*Thomas L. Isenhour (Department of Chemistry, Kansas State University, Manhattan, KS 66502, USA)*

The goal of the Analytical Director project is to build a system that can design, test, modify and implement its own analytical procedures. The Analytical Director is a combination of commercial robotic and other laboratory equipment with computer based artificial intelligence. The prototype system is now able to design an entire analytical procedure in a high level language working from an icon driven windowed display and have the robot laboratory execute that procedure without human intervention.

### Flexible, intelligent systems for automated chemical analysis

*Frank A. Settle, Jr. (Department of Chemistry, U.S. Air Force Academy, USAF Academy, CO, USA), H. M. Kingston (Department of Chemistry, Duquesne University, Pittsburgh, PA, USA) and Michael Pleva (Department of Chemistry, Washington and Lee University, Lexington, VA, USA)*

Commercial expert system shells, spreadsheet and database programs can be combined with automated laboratory equipment to form flexible, intelligent systems. After a brief introduction, three examples were presented. The first system integrated an expert system shell, VP-Expert, with a Zymark System V robot to perform qualitative analysis for selected metal cations. The second system currently under development, combines VP-Expert, Lotus 123 and an experimental design program with the Zymate System and a Milton Roy spectrophotometer for the design and implementation of colorimetric phosphate determinations. The final system involves the PC-Plus shell and dBase III linked to a Zymark robot and a CEM microwave unit to perform dissolution of a variety of sample matrices.

### Encapsulation and transfer of standard methods using automated equipment

*H. M. Kingston, Peter Walter (Duquesne University, Pittsburgh, PA, USA), and Frank A. Settle, Jr. (Department of Chemistry, U.S. Air Force Academy, USAF Academy, CO, USA)*

Microwave sample preparation is gaining a wide degree of acceptance within the analytical community. The Environmental Protection Agency (EPA) is in the process of approving two microwave dissolution methods for trace elemental analysis (Methods 3015 for aqueous samples and 3051 for soils, sediment, sludges, and oils). Required procedures such as these provide solid rational for automation and new challenges for accurate methods transfer. The nature of standard procedures requires that they be readily transferable and reproducible between laboratories. Microwave sample preparation offers two viable methods for transfer of microwave conditions: equipment calibration and feedback control.

Calibration of microwave equipment permits transfer of procedures between various models and manufacturers by replicating the microwave field flux of the equipment. Feedback control of microwave equipment is based on real-time measurement of temperature and is also a viable way of transferring the test conditions.

Until recently, procedures were transferred between analysts using mainly manual equipment. With the development of automated equipment, a new aspect of method transfer must be addressed – the electronic transfer of analytical chemical procedures. Three automated microwave sample preparation systems have been designed and are located at EPA sites in Las Vegas, Nevada and Seattle, Washington, and at the National Institute of Standards and Technology (NIST, Gaithersburg, MD). Methods to capture the environmental procedures are being evaluated and tested with these automated systems. The encapsulation system combines a microwave procedure generation, sample log-in, calibration, and microwave control program, and various versions of system hardware control software. The hardware consists of an integrated Zymark robot with peripheral equipment, System V controller, and a PC clone to control the automated microwave equipment. The microwave procedure generation program creates a procedure file that includes information required for a completely manual operation or for control of a fully automated system. The encapsulation of the method into a computer file is the basis for procedure transfer between these automated digestion systems. The two EPA sites are currently running routine samples using this method of encapsulation and rely on calibration to transfer the microwave conditions.

This example was used to discuss these specific methods, as well as the general topic of encapsulating analytical procedures and their electronic transfer to automated systems. It may soon be possible to down-load analytical chemical methods in the same way programs are transferred today.

### Objected-oriented data handling system for an automated chemistry laboratory

*P. A. Medvick, S. M. Mniszewski and T. J. Beugelsdijk (Los Alamos National Laboratory, Los Alamos, NM, USA)*

The environmental-remediation efforts at DOE complexes require characterizing problems at each site before taking clean-up action. Characterization will require the chemical analysis of millions of samples at a significant cost. Automation of the required chemical analyses methods provides a cost-effective solution. Five of the Department of Energy (DOE) national laboratories, Los Alamos National Laboratory (LANL), Pacific Northwest Laboratory (PNL), Sandia National Laboratories (SNL), Idaho National Engineering Laboratory (INEL), and Lawrence Livermore National Laboratory (LLNL) have joined together to automate the environmental chemistry laboratory. An object-oriented approach was deemed necessary to allow for modularization, maintainability, reusability, and flexibility of the software and hardware.

Each chemical analysis method is implemented as a Standard Analysis Method (SAM). A SAM is, in essence, a 'black box' into which a sample enters at one end and chemical or physical data 'exists' at the other. A SAM is composed of a set of Standard Laboratory Modules (SLMs) implementing sample preparation operations (such as weighing, grinding etc.), analytical instrumentation operations (such as measurement control etc.), and data interpretation operations such as (data reduction and documentation). Each of the SLMs are modular, standardized, and highly automated.

The software system is being developed in C++ building upon existing code developed by SNL for a Waste Storage Tank Generic Controller. The C++ based, object-oriented database, ONTOS by Ontologics, is being used to store the knowledge for operating the automated laboratory as well as the information collected from the sample analysis. ONTOS provides a graphical tool for database construction, C++ library routines for programmatic interaction, ONTOS SQL for queries within application programs, and a tool for visually and interactively creating graphical user interfaces for database access.

Database objects created were selected for quality control, hardware set-up and control information, and data information. The ADISS constructs by R. Lysakowski were used as a basis for the initial database design, which was subsequently modified to conform with the SAM system requirements. The following major objects were identified for this database: administrative, sample, aliquot, physical component, SLM, SAM, supplies, operation-report, status, calibration-results, raw-data, and processed-data. This knowledge, its structure, and how it is used to drive the system were further explored in the paper.

### MICROPLATES

### Fluid handling evaluations for microplate assays

*Gerald Hahn (Beckton-Dickinson Advanced Diagnostics, Baltimore, MD, USA)*

One of the most important aspects of an automated microplate system are the fluid-handling components. These are usually stepper motor driven syringes operated through a control interface. The accuracy and limitations of these fluid handling instruments will be discussed. The Zymate modules and the Tecan 505 system were compared. Improved user modules and programming suggestions were also discussed.

### The microplate robotic system as a complex biochemical and immunochemical detector for high throughput chromatographic analysis of serum lipoproteins

*Dennis S. France, Naved A. Surve, Robert E. Quinby, Mary E. Russell, Mary Kae Murdoch, Kathy Ramos, James R. Paterniti, Jr. and David B. Weinstein (Department of Lipid & Lipoprotein Metabolism, Sandoz Research Institute, East Hanover, NJ 07936, USA)*

While robotic systems have been frequently interfaced to HPLC systems, they usually play the role of automated sample preparation prior to chromatographic analysis. These roles have been reversed in a system in which a Zymate II Microplate Management System serves an HPLC system in a variety of functions. The prototype of this system has been previously described with one Pharmacia Superose-6 FPLC column and one autoinjector. The pivotal components of the current system are four FPLC columns, two Gilson 203 fraction collectors, two Waters WISP autoinjectors, and two Waters WAVS automated valve switching stations. In addition to replacing fresh microtube racks into the fraction collectors, the robot performs a number of lipid and apoprotein ELISAs on the fractions and then stores the fractions for further analysis. Despite the increased throughput of 96 complete lipid (cholesterol, phospholipid, and triglycerides) and apoprotein (apo A-I, B and E) profiles in a 24-hour period, the robot arm was still idle nearly 70% during a robotic run. This idle robot time has been exploited by splicing multitasking clinical chemistry analyses previously run by a different robot into the top level FPLC application program. Two major innovations have made this possible. Firstly, liquid handling programs involving the multipipette hand have been streamlined and optimized to allow variable volume aliquoting of serum samples into various replicate assays. Secondly, we have utilized the force feedback potential of the gripper hand to simply look for sample plates during the robot's idle time while serum samples are fractionating and collecting. Analyses are initiated when the robot perceives that the samples have been provided. Preliminary data will be presented regarding both the Zymate XP arm on this system and novel applications by the other robot, having been freed up by the FPLC robot, of high throughput cell-based drug discovery screens.

### Modifications of robotic enzyme-linked immunosorbent assay (ELISA) system for rodent serology to enhance capacity, throughput and sensitivity

*William R. Shek, Paul A. Oskar and Mark A. Cerra (Charles River Laboratories, Wilmington, MA, USA)*

Adventitious viral and mycoplasmal infections of laboratory rodents can cause changes that alter research findings. Some viruses indigenous to rodents are zoonotic agents capable of causing disease in humans. Rodents are therefore routinely monitored for viruses by assaying for specific antibodies formed as part of the immune response to infection.

During the past year, the authors have switched from a partially automated ELISA procedure to one that is fully automated, using the Zymate II Microplate Management System. The results of the evaluation of the robotic system and comparison of its sensitivity and specificity to those of the non-robotic procedure were recently published (Cerra *et al.*, LRA 2:119–131, 1990). In all aspects, including accuracy and precision of pipetting stations, and assay sensitivity and specificity, the fully automated robotic system compared favourably with the partially automated ELISA.

Since that publication, modifications have been made to the robotic system to further enhance its performance. The Zymate II controller was replaced with a System V controller which (in contrast to the former) is able to read and write ASCII files. With the Zymate II controller, essential array variables were sent from a remote PC-XT to the robot via a Z845 interface. With the System V controller, this slow step has been eliminated. Instead, the array variables, written to the files by the remote computer, are read directly from the controller disk drive. This is done at the beginning of each assay cycle so that a run can be started before all sample and test plates have been loaded. As a consequence, robot runs can be started earlier in the day, thereby increasing the actual daily throughput. The standard Zymark microplate storage rack holds 15 microtitre plates. These have been modified to hold an additional 5 plates per rack. By adding a 6th test plate storage rack, we have increased the maximum number of test plates per run from 75 to 120. An additional 10-slot incubator has been placed on the robot table to double the test plates processed per assay cycle from 10 to 20 and the incubation period per step from 20 to 40 min. This longer incubation has increased assay sensitivity. Other modifications that have improved system performance and reliability were also discussed.

### Automated affinity column chromatography system

*Kirk Andree, Dana Branham, Sandy Marshall (Hamilton Company, Reno, NV, USA), Martin Gross (Isolab, Inc., Akron, OH, USA) and Robin Felder, Ph.D. (University of Virginia, Charlottesville, VA, USA)*

The automation of assay processes can make significant contributions to throughput and assay precision, whilst reducing labour costs in most laboratories. Robotic pipettors have traditionally been used exclusively for the automation of the pipetting in clinical pharmaceutical and biotech laboratories. The system presented applied the robotic pipettor not only to pipetting, but also to the full automation of an affinity chromatography method based on a gravity feed, disposable column. The approach using the (Glyc-Affin) GHb (Isolab, Inc.) assay for total glycated haemoglobin in whole blood was described.

A Microlab 2200 robotic pipettor (Hamilton Company) was equipped with special hardware which automates the steps involved with fractionating glycated haemoglobin from non-glycated haemoglobin. The manual procedure is labour intensive and can be error prone; it requires several pipetting steps, extended column drain periods, mixing, dilutions, and critical timing steps. As the number of samples and columns to be processed increases, the robotic system remains consistent, where the possibility for procedural error increases with the manual approach.

The automated system limits manual intervention to approximately 30 min for assay set-up to load samples, columns fraction collection tubes, microplates and reagents. After set-up the technologist can walk away for the duration of the procedure (22·5 h). The robotic pipettor takes the whole blood sample and processes it through to the final fractions in microplates. Assay CVs are reduced from 7–10% manually to 2–3% with microlab Glyc-Affin, due to precision liquid handling, timing and column manipulations. Total operator time per 96 samples is 30 min with the automated system versus 3 h manually. Data were presented comparing the assay performance and operator time for the manual method and the automated system. Alternative applications, including solid phase extraction and filtration were explored and presented.

### MANAGING LABORATORY AUTOMATION

### The laboratory of the 90s – planning for total automation

*Linda A. Brunner (Ciba-Geigy Corporation, Development Department, Pharmaceutical Division, Ardsley, NY, USA)*

The analytical laboratory of the 1990s must be able to meet and accommodate the rapid evolution of modern-day technology. One such area is laboratory automation. Total automation may be seen as the coupling of computerized sample tracking, electronic documentation and data reduction with automated sample handling, preparation and analysis, resulting in a complete analytical procedure with minimal human involvement.

This is a goal that many of us, in various industries employing analytical technology, are working towards as we enter into the 90s. However, the requirements may vary from one laboratory of facility to another. Therefore, the automation has to be flexible enough to cover a wide range of applications, and yet fit into specific niches depending on individual needs.

Total automation must be planned for, well in advance, if the endeavour is to be a success. Considerations must be made for available space, laboratory layout, proper equipment, and the availability and access to necessary utilities. Adequate training and experience of the personnel working with the technology must be ensured. In addition, responsibilities of installation, programming, maintenance and operation have to be addressed. Proper time management and the efficient implementation and use of total automation are also crucial to successful operations.

The author's insights into laboratory organization and requirements, as well as management issues that must be faced when automating laboratory procedures were discussed.

### Measuring the effects of laboratory automation

*Gerry Fitzgerald and Jim Swanson (SmithKline Beecham Pharmaceuticals, Research & Development Division, King of Prussia, PA 19406, USA)*

A variety of scientific and management motivations lead to the automation of laboratory systems. Assessing the impact that automation has had on the organization is a part of maintaining these systems. SmithKline Beecham R&D is using several different types of measurement and many different tools for measuring the effects of automated laboratory systems. These metrics show how automated laboratory systems are affecting the workflow and information flow in each laboratory. This targeted program of metrics has increased management confidence in laboratory automation efforts, helped anticipate data processing bottlenecks, and highlighted end-user support needs. These metrics have been automated themselves wherever possible.

### Strategic planning for bioanalytical automation: managing growth successfully

*Julie J. Tomlinson (Glaxo, Inc., Research Triangle Park, NC, USA)*

Bioanalytical Automation rapidly expanded at Glaxo Inc. through the first half of 1990. During that growth period it became apparent that to continue to be successful, the growth of the technology and the resources required it must be planned and managed. A strategic plan was prepared to determine where we are, where we are going, and what we need to do to get there.

The plan describes the goals of the Bioanalytical Automation Group and the most important requirements for achieving those planned goals:

(1) Long-term management commitment.

(2) Trained, dedicated personnel.

(3) Quality facilities.

(4) Teamwork.

(5) Investment in automation-compatible equipment.

The strategic plan was a year old at the time of this presentation. The progress of the plan was discussed.

### Managing robotics in the generic pharmaceutical arena

*Marianne Scheffler (Danbury Pharmacal, Inc., Carmel, NY, USA)*

Danbury Pharmacal introduced robotics in 1987 as a way in which to optimize throughput, reliability, and cost. Through the company's experience with robotics, this paper gave an overview of various issues, such as acceptance by upper management as well as on the chemist level, political confrontation, validation processes and product throughput.

### Selection criteria for laboratory robotic application personnel

*Peter W. Rulon (Advanced Technology and Validation Section, Quality Assurance Department, Norwich Eaton Pharmaceuticals, Inc., Norwich, NY 13815, USA)*

Norwich Eaton Pharmaceutical recognized the benefits of using automation systems in the laboratory over seven years ago and created a robotic development area within the analytical method development group. Since that time, they have placed into routine operation eight complete robotic systems and a large number of semi-automated systems.

This level of activity has provided many challenges for the automation group. The success of this group has been very dependent on the number of talents of people working these assignments. You can have the best equipment and the vendor's promises of success, but our experience has shown over and over again, it is the people who understand our products and our requirements that get systems on line.

Assembling an effective robotics organization requires prework on the part of management. There must be a clear vision of the specific types of activities the group will perform. This vision can be used to establish a skills profile for the members of the team. The size of the team is also important to assure momentum towards success. The author's experience suggests at least four people are required to provide the variety of skills and keep the group going.

Each member's personality type is an important component of establishing a new team. In robotics, one of the most critical talents is the ability to work on long-term projects that constantly present new challenges. The group members need to balance consistency of purpose with the ability to creatively solve a variety of problems. The group will not be effective in delivering new technologies unless they have the talent to train the novice in a highly technical environment.

People who are successful in automation development are unique. They should have the ability to work comfortably in a logic based environment, to become very creative on demand, and to communicate highly technical information effectively. People do not usually possess all these skills, providing their manager with challenging coaching opportunities.

### Helping laboratory automation live up to its promise

*Gary W. Kramer (National Institute of Standards and Technology, Chemical Science and Technology Laboratory, Gaithersburg, MD, USA)*

The demand for chemical analyses is growing at an exponential rate. However, the advancement of laboratory automation is progressing, at best, linearly. Why? We all want to be able to answer analytical questions better, faster, cheaper, more safely, and with less personal drudgery. Laboratory automation has long promised to deliver such benefits. But building automated systems is still too difficult. Although some progress has been made, the goal of totally automated analyses and laboratories remains tantalizingly distant. How can we make the creation of automated analyses simpler, less expensive, and less risky to undertake?

Standards can help. To interconnect chemical analysis equipment to form automated systems, we need to minimize the amount of custom development required. Standarizing the manner in which we control instrumentation and the ways in which we move data, samples, and materials through the system can greatly facilitate the fabrication of automated systems. Adherence to such standards will allow the creation of automated building blocks that can be easily interconnected to analytical systems.

Another impediment to the development of laboratory automation is the current shortage of people with the requisite skills to specify, design, create, install, modify, customize, update, and maintain such instrumentation. Expertise in chemistry (or biochemistry), mechanical engineering, electrical engineering, and computer science is required. Since it is rare to find individuals with this complete skill set, larger organizations have set up groups to handle automation development. Smaller operations, which cannot afford the luxury of such a multi-talented group or find an automation Renaissance man, face a struggle in their automation efforts.

The Consortium on Automated Analytical Laboratory Systems (CAALS) was created at NIST to foster the development of laboratory automation. Its programs, including efforts to establish automation standards and to augment the pool of automation practitioners, are being supported by leading users and manufacturers of laboratory automation. By working together on problems that cannot be solved by a single organization alone, we plan to bring the benefits of automation into the laboratory more quickly.

---

### BIOLOGY/BIOTECHNOLOGY

### The use of robotics in a cosmetic microbiology laboratory

*Steven Schnittger (Estee Lauder Research & Development, Melville, NY, USA)*

The preservative challenge test is used to determine the efficacy of the preservative system in a cosmetic formulation. This method is a highly labour intensive, repetitive task, which utilizes many man hours. For this reason, a robot was developed to perform this function and free the microbiologist to perform other functions.

### The isolation of unique microorganisms from soil communities

*Lynda Ford, Dan Bowden, Heather Boll, Walter Raas, Joe Lykins, Jacob Zynger, Linda Bergin, Quensetta Lucas and Otis Godfrey (Lilly Research Laboratories, Eli Lilly & Company, Indianapolis, IN, USA)*

The screening of microbial colonies for the production of new pharmacologically active compounds is a labour intensive, integrated process consisting of colony isolation, fermentation, sample preparation and assay. In the flex #1 workstation a Mitsubishi Movemaster EX Robot weighs, sonicates, dilutes, and delivers individual soil samples onto a series of different agar-containing plates. Following incubation and colony growth, the plates are scanned by the vision workstation. An ASCII file containing colony measurements and locations is processed by a fortran program which eliminates bacterial contaminants using rules relating their size, % light transmission and uniformity of reflected light. Rules for pattern recognition are then employed to eliminate replicate colonies from each soil sample. In addition, rules for the selection of rare colonies are generated after analysis of data files stored in an S1032 database on the Vax mainframe computer. The selected colonies are then transferred into a module containing fermentation medium by a Puma 560 Robot (previously reported at ISLR 1988). After incubation, the flex #2 workstation adds methanol to each vial and transfers aliquots of the broth into a large number of microtitre plates. These plates are subsequently assayed for the presence of active compounds. Additionally, the flex workstations can generate genomic libraries in *E. coli*, purify plasmid DNA and transform suitable biophores.

### Automated procedures for the determination of enzymatic activity

*Richard Harrison and Maria Izquierdo-Martin (Merck Sharp & Dohme Research Laboratories, P.O. Box 2000, RY-80N-A58, Rahway, NJ, USA)*

Kinetic characterization of enzymatic activity has long been a labour intensive and time-consuming process. A fully automated robotic system was designed for the kinetic evaluation of enzymatic activity. The robotic system is a standard Zymate robot interfaced to an HPLC. Three unique features were incorporated into the apparatus; a low dead volume HPLC interface was designed which minimizes the loss of precious, expensive reagents, a device which allows solutions of temperature sensitive biological samples to remain accessible on the work surface at 4 °C, and a temperature controlled rack which keeps reaction mixtures within 0·1 °C of the set temperature.

With this robotic system, it was possible to determine kinetic constants for several enzymes. To illustrate the utility of the robotic system, kinetic evaluation of the metalloprotease Stromelysin was presented. Standard deviations of initial velocities measurements are lower than when performed manually. The kinetic constants $K_m$ and $V_{max}$ are defined more precisely than with a previously described manual method (*Anal. Biochem.*, **180**, 1989, 110), at one fifth the time. This is the first reported fully automated chromatographic system capable of determining enzymatic kinetic parameters.

### Automation for mapping of the human genome

*Mary M. Blanchard, David Sloan and Volker Nowotny (Washington University School of Medicine, St. Louis, MO, USA)*

With the biochemical prerequisites at hand, the problem of establishing a map of the human genome that consists of about 3 billion basepairs was tackled. First, the human DNA is kept in a library consisting of yeast clones, specifically, Yeast Artificial Chromosomes (YAC). Each YAC contains a piece of human DNA up to a length exceeding a million basepairs. This procedure allows for the maintainance and propagation of the human DNA. Second, the Polymerase Chain Reaction (PCR) amplifies short tracts of specific DNA from within a very complex mixture of DNA sequences, making only this specific DNA visible. Thus, through the use of PCR, it is possible to recover a clone from the library which contains the

DNA sequence which is of interest to the investigator. In order to implement PCR in the screening of the library, the DNA of all the clones must be purified and arrayed in a pooling scheme. Then, one may identify positive clones through a binary screen of this scheme.

Mapping of the human genome or human chromosomes thus becomes the repeated screen of the library for defined sequences (Sequence Tagged Sites; STS). Screening data will provide information about the order of the clones, thereby generating a chromosomal map. For a sufficiently dense (on average one STS each 50 000 basepairs) coverage of the genome, about 3000 STS's per chromosome are necessary.

To automate the biological experiment (PCR), we utilize a robotic workstation that is based on the BIOMEK 1000 system (Beckman Instruments, Palo Alto, CA 94304). Necessary modifications involve the increase of available tip boxes to the robot's envelope, the design and building of a thermocycler capable of handling 576 samples per round and a storage unit to have access to DNA samples of about 60 000 yeast clones. The major effort involves the assembly of software for the control of this machine to allow a completely automatic screen integrating thousands of pipetting steps and the transfer of hundreds of micro titre trays each day.

### ENVIRONMENTAL

### Laboratory robotics applications in environmental research on the fate of pesticides in soil

*William C. Koskinen, LeEtta J. Jarvis and Robert H. Dowdy (USDA – Agricultural Research Service, and Soil Science Department, University of Minnesota, St. Paul, MN, USA)*

Characterization of the environmental behaviour of pesticides in soil entails field studies and laboratory experiments under controlled conditions. Field experiments to determine the dissipation and movement of pesticides and laboratory experiments to determine the effect of environmental factors such as soil temperature and moisture on pesticide degradation often require thousands of samples to be analysed. The analysis is a multi-step process including: extraction of the pesticide from soil, sample clean-up and preparation, identification, and quantification. The extraction of pesticides from soil and subsequent sample preparation is the limiting step in the analytical procedure. A robotically automated procedure has been developed by the authors for the extraction of a variety of herbicides from soil and subsequent sample preparation. The procedure includes: adding extraction solvent to soil; mixing the soil-solvent suspension; centrifuging the suspension; removing supernatant solution containing pesticide from soil; evaporating supernatant down to water; absorbing pesticide remaining in water onto solid sorbent; stripping pesticide from solid sorbent; placing final pesticide solution into vial; and capping the vial for GC or HPLC analysis. With serial processing of samples by the robotic system, as opposed to manual batch processing sample throughput increased by a factor of three, labour decreased by half and precision of results significantly improved. The same basic laboratory robotic system is currently being used in other types of pesticide-soil research. For instance, the batch equilibration method used to characterize pesticide adsorption-desorption was automated This method involves equilibrating an aqueous pesticide solution with soil for a period of time, separating the solution from the soil, adding solution without pesticide to the soil and repeating this process a number of times. After each step, the pesticide is extracted from each solution and analysed. The experimental variables are soil type, pesticide solution concentration, aqueous solution composition, and equilibration time. As part of a typical adsorption-desorption experiment, soil samples (three soil types, four pesticide solution concentrations, three replicates) are processed after successive 3 h equilibrations over a 72 h period. The experimental procedures for the elucidation of binding mechanisms of pesticides to soil and the optimization of parameters for extraction of pesticides from soil have also been automated.

### Zymark robotic automation of EPA Method 505: analysis of organohalide pesticides and commercial polychlorinated biphenyls (PCB) products in water by microextraction and gas chromatography

*Russell M. Spencer, Joseph A. Poland (CT Dept. of Health Services Lab Bureau, Hartford, CT, USA), and Marsha A. Paul (Zymark Corporation, Hopkinton, MA, USA)*

Zymark robotics are uniquely qualified for environmental water testing through the efficient automation of EPA Method 505. This method consists of extracting 35 ml of a water sample with 2 ml of hexane. Aqueous calibration standards are extracted and analysed in parallel with the samples in order to compensate for extraction losses. A single injection of 4 μl of the hexane extract is made into a gas chromatograph with one injection port split to accommodate two different capillary columns utilizing dual linearized electron capture detectors.

Zymark automation performs all the required laboratory steps of the method completely and consistently from sample acquisition to final analyte identification, confirmation and quantitation. The Zymark PyTechnology system is composed of a concentric ring of specific functional modules serviced by a central Zymate II robotic arm. Laboratory processes including aspiration, weighing, mixing, extraction, transfer and injection into the gas chromatograph have been integrated into a fully automated system under command of the System V Controller.

Certification and validation of the system was achieved by multiple analyses of proficiency sample matrix spikes supplied with EPA Water Pollution Study Number WP023. Seven different pesticides including aldrin, dieldrin, pp-DDD, pp-DDE, pp-DDT, heptachlor, and heptachlor epoxide were analysed. Follow-up studies are in process for 14 additional pesticides and 7 PCBs. Linear regression curves plotted for aqueous multilevel extracted standards (7 levels) yielded a correlation equal to or better than 0·9997 for the above pesticides over a concentration range of 0·04 to 30 ppb in water. Final concentration results for eight replicate analyses of each of these compounds all fell within EPA Acceptance Limits. Mean per cent recovery was 92% or better of the true values for five pesticides, the exceptions being heptachlor at 84% and dieldrin at 88%. Other statistical considerations include a standard deviation range of 0·02–0·08 and a variation from 42–11 for these compounds.

Significant savings in terms of laboratory resources are realized when comparing the Zymark Automation System with manual procedures. Automated analysis of 40 water samples requires approximately 40 min to set up and run on the robotic system as compared to 240 min to complete manually. This is a 600% performance efficiency rating for the Zymark system. In addition, automation provides for completion of the assigned task during normally non-productive hours in the evenings and on weekends and holidays. In conclusion, reliability, sensitivity, precision and accuracy of pesticide analysis by EPA Method 505 combined with 600% greater efficiency over manual extraction techniques are the highlights of the Zymark Automation and PyTechnology.

### Automated environmental extract clean-up with the BenchMate workstation

*Richard A. Kern, Kevin O'Mara, Shirley Yaikow (Midwest Analytical Services, Detroit, MI 48201, USA), Louis J. Cercone, Eric Andersen, Lisa Moore (EnviroTest Laboratories, Inc., Newburgh, NY 12550) and Mark Cava (Zymark Corporation, Hopkinton, MA 01748, USA)*

Traditional extract clean-up methods require manual dilutions, filtrations, injections, column conditioning, and reagent additions. This paper presented:

(1) The automated clean-up methods.

(2) The machine-generated audit trail of volumetric accuracy and precision.

(3) The GC/MS results for the analytes of interest.

Two different applications of the BenchMate were examined: the traditional approved SX3 Bio Beads (EnviroTest); and the new high pressure column (Midwest Analytical Services).

Environmental extracts were analysed for semivolatiles, pesticides, and PCBs in waste oils. The BenchMate Workstation performed weighings, dilutions, filtration, and injection for GPC clean-up, florisil cartridge clean-up, and acid clean-up.

Fast turnaround, good data, and low cost, are just a few of the benefits that have been realized for these analyses.

### Design and development of a robotics based acid digestion system for environmental sample preparation: evaluation and characterization of acid digestion procedures for graphite furnace AA and ICP analysis

*Iqbal Dawood Tabni, Gray Ward (Enseco Inc., Quality Assurance and Health & Safety Division, Somerset, NJ, USA), Debra Hasford, Erik Natkin and Maxine Pitard (Enseco-Rocky Mountain Analytical Laboratory, Denver, CO, USA)*

A Zymate II laboratory robot system was used to completely automate the acid digestion procedures for metal analysis. The Robotics Based Acid Digestion Processor was utilized to prepare aqueous and soil samples for Graphite Furnace AA and ICP analysis. The salient features of this application are complete automation of acid digestion procedures for:

(1) Different matrices.

(2) Analysis type.

(3) Test type.

In this report the feasibility of a completely automated acid digestion procedures based on a robotics system for routine use was characterized and evaluated. A comparison of analytical data obtained from the manual procedures and automated system was presented.

### Robotic system for aqueous environmental sample preparation

*M. J. Zoellner, H. W. Emmel and L. D. Nelson (Dow Chemical Company, Midland, MI, USA)*

A robotic system for the automated preparation of aqueous environmental samples has been successfully implemented in the author's laboratory. The system is designed for preparation of aqueous sample types: river and well waters, surface waters and effluents. The sample preparations which are possible include digestion and/or concentration that follows EPA-600/4-79-020 methodology for trace elements via Inductively Coupled Plasma (ICP); the determination of arsenic and selenium via hydride AA; and the determination of mercury via cold vapour AA.

Heating the sample either for digestion or volume reduction purposes has been traditionally done using hot plates requiring almost constant attention to prevent any one sample from going to dryness which would volatilize any metals present thus rendering the analysis useless. It is this unpredictable non-uniform heating by hot plates that makes it a poor choice for automated sample preparation. The approach taken for this application was to use a water bath for heating the sample. The water bath provides for a uniform 98 °C heat applied to all the sample tubers which minimizes sample bumping and reduces the chance of cross contamination. This arrangement also eliminates the need to constantly monitor the evaporation progress allowing for unattended use. Since the procedure is a timed event, the program control allows for the added capability to use the robot arm for other purposes such as a serial dilution procedure while the samples reside in the water bath.

A high temperature heating block (300 °C) is used for the As, Se preparation procedure to allow the samples to be taken down to a fuming acid condition. The tubes are transferred to a fan cooled steel rack and allowed to cool to room temperature before returning to the final sample rack. This minimizes any accidental exposure to hot acid-fuming tubes since the robotic system is designed to operate utilizing a standard laboratory fume hood.

### Development of laboratory robotics at FDA: automated system for the determination of natural toxins and pesticides

*Allen S. Carman, Jr., Shia S. Kuan (Natural Toxins Research Center), Kenneth V. Miller and Humberto G. Guerrero (District Laboratory, U.S. Food and Drug Administration, 4298 Elysian Fields Avenue, New Orleans, Louisiana 70122, USA)*

Because of the reprogrammability of robot automated tasks, this approach to automation has been called flexible automation. This feature makes this type of automation particularly attractive to laboratories such as those in the FDA requiring increased productivity, but hindered by a frequently changing and often unpredictable, heterogeneous workload. To evaluate the potential of flexible automation, the Natural Toxins Research Center (NRTC) developed a workstation using a Zymate I System for the HPLC determination of solanaceous alkaloids in potatoes and the determination of aflatoxins in milk and other matrices. This system was described with emphasis on a column liquid level sensor whose development was critical to the successful completion of the aflatoxin method and an HPLC injector developed in-house to save costs. Complete changeover of this system from glycoalkaloid to aflatoxin assay can be accomplished in less than 2 h.

A second workstation, using a PyTechnology System, is being used to automate a pesticide residue screening procedure developed by the District Laboratory specifically for automation and the aflatoxin procedure described above. Comparison of recoveries of 15 pesticides by this procedure with a well-established screening procedure shows that this procedure compares favourably with the established procedure. The screening procedure and the robotics system were described. The design and construction in-house of a pneumatically-operated transfer arm, and the modification of a Zymark evaporation station to provide controlled evaporation were also presented.

### Single lab evaluation of a robotic microwave digestion system for preparing aqueous samples by Method 3015 and solid samples by Method 3051

*Daniel C. Hillman, Piotr Nowinski (Lockheed Engineering and Services Company, 1050 East Flamingo Road, Las Vegas, NV 89119, USA) and Larry C. Butler (Environmental Monitoring Systems Laboratory, U.S. Environmental Protection Agency, Las Vegas, NV 89114, USA)*

A robot system has been developed to perform microwave digestions of environmental samples. The system is comprised of a Zymark robot with several custom PySections and a CEM microwave digestion oven. National Institute of Standards and Technology (NIST) supplied the software to interface the CEM oven operation with the Zymark robot operation. The robot microwave digestion system automates one of the most labour intensive steps in inorganic analysis. In a conventional manual digestion procedure, analyst attention and input is required throughout the process, from weighing samples, adding reagents, monitoring the digestion progress, performing final dilutions, to transferring digestate sample bottles or instrument autosampler vials. Critical throughout a manual procedure is accurately recording the digestion data. With the robotic microwave digestion system, operator input is limited to adding sample to digestion vessels and transferring digestate to storage containers after digestion. All digestion steps (reagent addition, matrix spike addition, reagent blank preparation, loading and unloading the microwave oven, and final dilutions) are performed by the system. Digestion protocols are downloaded from a data file containing the protocol data. By using barcode labels and balances interfaced to the system, all digestion data is recorded automatically in a text file. The operator is not required to record any data.

Currently, two systems are being evaluated. One system is located in Las Vegas, Nevada and the other in Manchester, Washington. The primary difference between the systems is in the digestion vessel. The Las Vegas system uses the single-wall Teflon digestion vessel while the Manchester system uses the newer double-wall Teflon-lined digestion vessel. The Manchester system also further automates the process by dispensing sample (after digestion and dilution) directly into vials held in an ICPES autosampler rack.

This paper described the evaluation of both systems to perform digestions of both aqueous and solid samples following SW846 Methods 3015 and 3051. Results included estimates of both within and between lab precision and bias. Also, general characteristics of the system were described (speed, reliability, data output).

### Automation of an acid digestion process for water samples

*Gerald L. Hoffman (National Water Quality Laboratory, US Geological Survey, Arvada, CO, USA)*

The present US Geological survey manual method of solubilizing trace metals associated with waterborne particulates is a mild acid extraction procedure. To automate this procedure, it is necessary to change the manipulations and the conditions of the acid extraction. These changes are made not only to automate the procedure, but also to lower the blank values for several of the metals determined in the acid digested water samples. It is important, however, for the automated procedure to statistically duplicate the same acid-extraction efficiencies for trace metals as the manual procedure, because of the large historical data base generated with the manual procedure. Because of these requirements, the quantity and type of acid (2·5 ml of concentrated HCl per 100 ml of sample) is maintained. Also, the use of Whatman* 42 filter paper to filter the acid digested samples is continued in the automated procedure. Major changes to the manual procedure are as follows:

(1) The acid is added directly to the sample bottle, instead of transferring the sample to a beaker and then adding the acid.
(2) The closed sample bottle is heated in an oven for 8 h at 65 °C, instead of heating the beaker on a hot plate at 90 °C for 2 h.

### Automation in pesticide residue analysis by a robot system

*R. Brennecke (Bayer AG, Business Group Plant Protection, Institute for Product Information and Residue Analysis, Monheim, Bldg. 6610, D 5090 Leverkusen, Bayerwerk, Germany)*

Generation of crop residue and environmental fate data is an important part of both the development of a new pesticide and the support of existing products. The development process spans a number of years and generates a large number of different samples, from crops to soil and animal tissues, all requiring analyses down to low levels, typically from 0·01 to 0·05 mg/kg. The demand for this type of trace level analysis has increased strongly over the last five years at the author's Institute.

For handling all the samples it was decided to automate the clean-up steps in residue analysis. More uniform and simplified residue methods have been developed first, because the existing methods varied greatly and were too complex for automation. A liquid-solid extraction on diatomaceous earth followed by an adsorption chromatography on silica gel was the resulting clean-up procedure, which has been proven to be useful in routine analysis with a large number of plant materials and beverages and also for different pesticides. The next step was the adaptation of this procedure to a Zymate II Laboratory Automation System. A description of the adaptation and the validation of this clean-up procedure by a robot system was given.

---

### CHROMATOGRAPHY SAMPLE PREPARATION

### A validated liquid chromatographic assay for CI-988 in human plasma with batch robotic sample preparation

*E. L. Johnson, K. Rutkowski, J. P. Hinton and D S. Wright (Parke-Davis Pharmaceutical Research Division, Warner-Lambert Company, Ann Arbor, MI, USA)*

CI-988 is cholecystokinin-B receptor antagonist which is currently under investigation as a potential antianxiety agent. Human studies will involve analysis of CI-988 levels in a few hundred to possibly several thousand samples. Batch robotic sample preparation will be used to increase total sample throughput as well as decrease analyst time.

The method involves batch robotic sample preparation with a Zymate Laboratory Automation system and a custom vacuum box to hold the solid phase extraction cartridges. Up to 144 individual cartridges can be prepared in a batch mode. Solvents are added to the cartridges via a liquid distribution hand using either a multi-valve FMI pump or the master lab module as the solvent source. Vacuum, controlled by the power and event controller, is used to aspirate solvents through the cartridges. A TurboVap LV was used to evaporate eluent from the cartridges prior to analysis by HPLC with fluorometric detection.

The method has been validated and is suitable for routine analysis of CI-988 in human plasma over the concentration range of 0·25 and 500 ng/ml. Sixty samples can be extracted in 55 min. The rapid rate of sample extraction allows a single robot to be used by more than one individual during a routine work day.

### Analytical chemistry in the toxicology laboratory: a versatile robotic sample preparation system

*R. M. Kannuck, D. A. Redder and P. T. Hardesty (E.I. DuPont De Nemours and Company, Haskell Laboratory for Toxicology and Industrial Medicine, Newark, DE, USA)*

Toxicology testing under EPA and FDA guidelines must be conducted according to published Good Laboratory Practices (GLPs). These guidelines generally mandate the verification of administered dose levels as well as confirmation of homogeneity and stability of test material in the dosing vehicle. The duration of toxicology tests may range from two days to two years, with samples being submitted in batches for analysis at varying intervals throughout each study. The variety of testing requires analysis for the same compound in completely different matrices (animal chow, methyl cellulose, organic solvent) over concentrations ranging from several per cent to parts per billion.

Such a dynamic environment does not seem at first glance to be a likely candidate to benefit from robotic automation, since there is no single assay or method that can be optimized once and then used respectively for large numbers of samples over a long period of time. However, because the total number of samples from all concurrent studies is large and there are many repetitive operations involved in the multiple methods that are supported, this sort of laboratory can still be amenable to the productive use of robotics.

Haskell Laboratory has developed a flexible system based on a Zymark PyTechnology System that can be reconfigured easily to implement methods that change on a daily basis. The system can perform the subsampling, extraction, filtration and dilution functions necessary

---

* The use of brand names in this abstract is for identification purposes only and does not constitute endorsement by the US Geological Survey.

prior to chromatographic analysis. Additionally, system evaluation routines and documentation necessary to comply with GLP guidelines has been incorporated.

### Comparison of workstations for liquid-liquid extractions

*J. K. Bolon and R. F. Arrendale (Drug Metabolism Department, Glaxo Research Institute, Research Triangle Park, NC, USA)*

Automated sample preparation was considered as a means of enhancing productivity. Three automated workstations (the Tecan 5000, the Waters Millilab and the Zymark BenchMate) were evaluated for use in a liquid-liquid extraction procedure to quantify G187084 {methyl-3-[4-methoxycarbonyl-4-[(1-oxopropyl)phenyl-amino]-[piperidine]propanoate}, an ultra-short acting opioid, from whole human blood. Levels of the opioid were determined by capillary GC/MS/SIM. Accuracy was determined by weighted linear regression analysis of the calibrated standards. The BenchMate, Millilab and Tecan 5000 had correlation coefficients of 0·9991, 0·9981, 09978, respectively. Precision was measured by the coefficient of variation ($r$) of the interpolated standard curve concentrations. The coefficient of variance at the 1 ng/ml level for the BenchMate, Millilab and Tecan 5000 were 10·7%, 8·3%, 50%, respectively.

### Medicated feed assay applications using a fully automated PyTechnology robotics system

*L. C. Erhart, L. J. Kostek, J. E. Curley, A. Grizzuti and M. P. Synder (Pfizer, Inc., Groton, CT, USA)*

The Analytical Research and Development Department of Pfizer Central Research has provided support to toxicology research by assaying medicated feeds to assure appropriate drug content during dosing. Historically, feed assay methods have been laborious, time-consuming and difficult when drugs are extracted from complex feed matrices.

To improve laboratory efficiency, several automated procedures have been developed for the analysis of drugs in rodent feeds. The robotic system performs all the necessary steps to determine the concentration of drug in animal feed. The robot tares sample containers to which an operator adds medicated feed samples. After determining the sample weight, the robotic sample scheduler manages the robot's time and motion according to pre-set parameters to efficiently handle up to eight samples at a time. Extraction solvent is added and the containers are shaken to extract the drug. Serial dilutions are made into individual tubes. The drug is separated from the feed matrix by liquid-solid extraction or fatty oil precipitation. The solution is filtered and injected onto an isocratic HPLC system.

Application of these procedures with drugs in medicated feeds is discussed. Although the robotic method is inherently slower than the manual method, unattended overnight operation allows the user to maximize productivity. Precision and accuracy of the robotic procedures are comparable to those obtained with manual methods.

### Automated sample preparation of whole blood for cyclosporin using the BenchMate

*William Holman and Robin A. Felder (The University of Virginia Health Sciences Center, Department of Pathology, Charlottesville, VA, USA)*

Cyclosporin A (CsA) is currently the most selective immunosuppressant used clinically for the prevention of graft versus host disease following organ transplantation. The narrow difference between renal toxicity of an excessive dose and inadequate immunosuppression by a low dose makes constant monitoring of patient blood concentrations a necessity. Since immunosuppression requires continuous presence of CsA, organ transplant recipients usually continue to take CsA for the rest of their lives.

High performance liquid chromatography (HPLC) is the method of choice for measuring CsA. However, HPLC is plagued by low throughput since many manual preparative steps must be performed prior to analysis. Alternative methods, which include several variations of manually performed or automated immunoassay have relatively high imprecision, interferences from metabolic products and tremendously high cost. Therefore, automation of CsA analysis by HPLC should result in an easy to perform cost effective method.

The Zymark BenchMate was used to automate much of the sample preparation involved in solid phase extraction of CsA. Whole blood specimens were collected from venipuncture into heparinized glass tubes. Samples were placed on the BenchMate in the specimen holding area and a C18 Bond Elut column was placed in the top of the sample tube. Specimen extraction from this point was handled automatically by the BenchMate. The sample was injected manually into the HPLC system for quantification.

Similar recovery of CsA was found when manual and BenchMate methods were compared for three levels of calibrator. Analytical imprecision was smaller for the BenchMate when compared to manual extraction. Within run precision was found to have a coefficient of variation (C.V.) of less than 8% at either concentration. Between run precision was determined by performing a BenchMate extraction on two specimens each day for 20 days. Between run precision was less than 9% C.V. Eighteen patient specimens were extracted either manually or using the BenchMate and the results compared by linear regression. There was good agreement between the manual and BenchMate extracted specimens.

The BenchMate is useful for reducing the manual steps necessary to perform HPLC analysis of CsA. The BenchMate has a throughput of 10 min per sample which would allow up to 48 samples to be extracted in an 8 h shift. Since extractions are performed serially by the BenchMate, samples can be added to the BenchMate as they are received in the laboratory obviating the need for cut-off times for analysis.

### A generic robotic sample processor for HPLC analysis

*L. J. Lorenz, E. C. Jensen, M. E. Hinshaw, D. A. Jackson and J. Zynger (Lilly Research Laboratories, Eli Lilly and Company, Lilly Corporate Center, Indianapolis, IN, USA)*

Laboratory robotics generally follow a course where a robotic system is designed to mimic a previously developed manual procedure. This presents a problem since most procedures vary. Therefore, a different system is needed for each application changes or ceases to exist.

An alternative approach for utilization of laboratory robotics requires the development of a general purpose robotic system. Applications are then developed to fit the robotic system. Such an approach allows for robotic systems which can handle a large volume number of different methods without alteration of the robotic system.

Such a system has been developed as a sample processor for HPLC. Today, many samples which have very limited solution stability are examined by HPLC. This poor solution stability limits the ability to use classical automated autoinjectors for servicing an HPLC instrument. A robotic system can overcome this problem by preparing the samples just prior to analysis.

The robotic system is designed to make use of commercially available devices where possible. The system will handle liquid samples when desired but is primarily designed to handle dry powders, capsules, or tablets. The system will process a dry sample which is presented preweighed in a test tube. A solvent is added and the material is put in solution through a series of shaking and sonication steps. The sample can undergo a subsequent dilution step with mixing if desired. The solution is then transferred to a syringe filter which filters the sample and also acts as the driving force to push the sample through selection valves to direct the sample to a specific instrument and finally through a sampling valve to inject the sample into the HPLC system. The robotic system incorporates appropriate relays and logic to start an HPLC gradient to autozero detectors, and to start data collection for the sample. The robot documents all key steps in the process and documents all error recovery attempts when a problem is detected.

### GENERAL

### Trials, tribulations, and triumphs in PyTechnology

*Bruce Kropscott, Jeanne Hugo (Dow Chemical Company, Midland, MI, USA), and Shoreh Shabrang (Manteq International Corporation, Midland, MI, USA)*

The Zymate Laboratory Automation system has gone through considerable evolution since its introduction at the 1982 Pittsburgh Conference. The first Zymate robotics systems were able to perform a limited number of laboratory unit operations (LUOs) such as weighing, pipetting, mixing and container manipulations. The Zymate I robots were easy to direct through a series of positions but each position on the table had to be manually taught. Each LUO involved moving through multiple positions and actual applications used hundreds of positions, thus applications were physically labour-intensive to set-up and took weeks even months to fully implement. The custom CPU controllers were limited in the amount of memory; had no inherent error detection; and LUO verification was left up to the individual programmer.

Since the first introduction, the robotics systems have been transformed through rapid improvements such as increased speed, tactile and axis sensing and new LUOs capabilities. In the 1990s, PyTechnology Robotics Systems have many pre-positioned peripheral modules with pre-programmed LUOs thus allowing a faster start-up time for standard applications. PyTechnology programming has extensive error checking to help the user to anticipate probable errors and has pre-determined routines to recover from those common errors. Unfortunately, many of our applications require custom modules; use more than the allotted number of available PySections; use a variety of containers; and incorporate non-standard routines for the pre-programmed LUOs modules.

This paper described (1) the advantages and constraints of PyTechnology for custom applications; (2) efficient use of the working space by stacking racks and combining LUO modules; and (3) basic philosophies and approaches for custom software in the authors' method development system.

### Automating test methods for evaluating chemical warfare protective materials

*Raymond E. Andreotti (U.S Army Research, Development and Engineering Center, Soldier Science Directorate, Natick, MA, USA)*

The US Army's research programme on the development of chemical protective clothing involves the evaluation of fibres, foams, films and powders for sorptive and/or reactive activity toward chemical warfare agents. Experimental samples are subjected to droplets or vapours of chemical agent simulants in closed containers for specific time periods. The sorptive and/or reactive characteristics of the sample are measured as a function of time by analysing for the remaining simulant using a gas chromatograph of a UV/visible spectrophotometer. Liquid-state tests are also performed utilizing a Fluoride-sensitive electrode to detect the hydrolysis product of the reaction. Due to the variety and complexity of the tests, as well as the toxicity of chemical agent simulants used, automation is a real challenge. This paper discussed how the flexibility of three laboratory robots was utilized in developing automated test methods that improved safety, accuracy, and productivity under hazardous testing environments.

### Pouring difficult solids under diverse conditions

*Stephen R. Metzner (Monsanto Agricultural Company, 700 Chesterfield Village Parkway, St. Louis MO 63198, USA)*

When working with solid materials, it is often necessary to pour all or part of the solid from one container to another. Often the weight of the solid is desired before dissolution, extraction or subsequent handling. The nature of solids may vary widely and contribute to the difficulty encountered when a robot pours the material.

The need to keep containers capped may control which containers are used to hold the solid. The nature of the receiving container may also be determined by subsequent analytical procedures.

Beginning with a powder pouring routine developed by Zymark, this poster presentation described the successes and failures of robotic pouring of difficult solid materials.

### Polymer ashing – a new look at an old technique

*Joe Cross, Ron Jones, Connie Lowe and Mitch Meyer (Phillips Petroleum, Bartlesville, OK 74004, USA)*

Ashing has long been a conventional technique for preparing polymers for analysis of catalyst residues and trace metal contaminates. It is a labour intensive, messy technique, used to prepare samples for both spectroscopic and gravimetric procedures. The conventional technique involves 30 to 60 min burning in a crucible over an open flame, followed by a high temperature ashing of the residue in a muffle furnace to remove all traces of carbon residue. The open flame burn-off requires operator attention and is a process that would be difficult to adequately automate. This report detailed the results of an investigation of an alternate technique for ashing polymers; direct ashing of the polymer in a high temperature muffle, in order to arrive at an acceptable procedure that eliminates open flame burn-off and simplifies automation.

### Designing automation for laboratories

*Michael A. Roos (Laboratory Robotics Company, St. Louis, MO, USA)*

Over the years of designing laboratory automation several lessons have been learned from systems that were successful, and equally important, from systems that failed to meet expectations. The automation designers that are most successful today have learned these lessons and are incorporating them into their system designs.

Several examples of design criteria and strategies were presented. The most important aspect of any design is the careful and realistic planning of the specifications and expectations. The next design step is the proper specification and selection of automation, and hardware. System design is shifting away from performing detailed tasks by the robot, toward parallel operations that use the robot as a facilitator between sophisticated workstations. Designs often require modifications of the hardware and/or modifications of the task. While compromises are often essential, they represent a risk for eventual dissatisfaction, and potential failure. A common compromise is giving up space in a crowded laboratory. Newer designs are becoming more compact and vertically integrated.

### Custom automation techniques

*Ron Hunt (Helene Curtis, Inc., Chicago, IL, USA)*

Bench-top automation to enhance productivity in the laboratory is, for the most part, available off the shelf. In some cases, however, parts of the application, or even all of the application require custom designed workstations. This presentation will discuss the advantages and disadvantages of in-house design versus contract vendor design. Examples were given on the handling of heavy wet samples by use of custom fingers, the implementation of barcode readers for decision and alignment purposes, and the mechanics/control of custom workstations.

---

### PLENARY

### Superior productivity – in the laboratory and beyond

*Francis H. Zenie (Zymark Corporation, Hopkinton, MA 01748, USA)*

Today, the need for more productive laboratories is more compelling than ever before. As we enter the 1990s, intense world-wide competition and increasing regulation challenge our laboratories to ensure: more precise results, unquestioned documentation, faster turnaround and better utilization of skilled people.

Superior productivity demands the greatest economic values from our resources; people, time and capital. Not only must modern laboratories improve their internal productivity, they must enhance productivity throughout the organization.

Many leading businesses have embraced laboratory automation as a strategic foundation. They invested to gain knowledge of new technologies and experience applying them – leading to impressive results. This presentation described specific case histories and drew powerful conclusions from the common elements of these real-world examples.

---

### POSTERS

### System data management for automated tablet assay workstations

*Jeff Roberts and Michael Shirley (Bristol-Myers Squibb, Products Division, 9707 Chapel Hill Road, Morrisville, NC 27560, USA)*

The authors' purpose was to design a data management system that would allow us to generate batch data summary reports, as well as to capture critical system data, in order to continually monitor a Customized Automated Tablet Assay Workstation via SPC Charting.

The critical data, from both sample preparation and subsequent HPLC Analysis, is captured real-time by the System V Controller and a PE Nelson Interface. The data is then imported into Lotus 1-2-3, and is manipulated by a series of Lotus Macros.

The poster showed sample programs written in EasyLab, PE Nelson Chromatography, and Lotus Macros software languages. In addition, sample data and graphs were presented.

### Automated extraction of XDE-436 from dog chow replaces soxhlet extractions

*Cynthia N. Peck (Analytical Chemistry Laboratory, 1803 Building, The Dow Chemical Company, Midland, MI 48674, USA).*

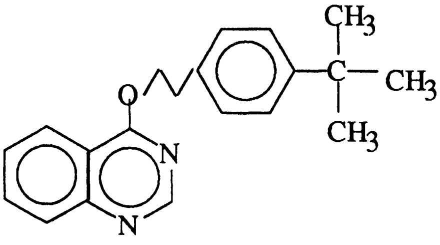

XDE-436

An automated procedure for the extraction of XDE-436, an experimental miticide, from dog chow has been developed using a PyTechnology robotics system. This procedure reduces the analyst time required for the extraction procedure from 6 to $1\frac{1}{2}$ h for 40 samples and eliminates the use of tedious soxhlet extractions to remove the test material from the feed.

XCE-436 (4-(2-(4-(1,1-dimethylethyl)phenyl)ethoxy)-quinazoline) is currently undergoing toxicity testing in dogs and rodents. Previously developed analytical methods determined that XDE-436 is stable in rodent and avian diets for up to 30 days.

XDE-436 could be quantitatively extracted from spiked samples immediately after mixing using a simple solvent extraction. However, the recovery dropped below 90% about 12 hours after mixing. The feed samples often arrived for analysis late in the day, preventing same day manual extraction without overtime work.

An automated method for solvent extraction of the feed, followed by centrifugation and dilution of the extracts was developed using a fully equipped PyTechnology System. The analyst can prepare the system for unattended overnight extraction in less than 1 h after the samples arrive, allowing the test material to be removed from the feed within 12 h.

### A highly automated fermentation extraction system

*C. K. Marschke, L. R. Carter and D. D. Gleason (The Upjohn Company, Kalamazoo, MI 49007, USA)*

The Upjohn Company's Chemical and Biological Screening unit was organized to collaborate with the company's discovery units in exploiting natural products as sources for new therapeutic activities. The system described here provides for the processing of large number of microbial fermentation broths and extracts.

A major need on one project was to extract each fermentation broth with an organic solvent. The system to do this was designed in collaboration with our Laboratory Automation Support group. The micro-organisms were grown in specially designed, 45-well cassettes and a Zymark laboratory robot performed the extractions. This system has many unique aspects but most noteworthy is a multifunctional 'hand'.

The first step in the extraction procedure is the sequential addition of acetone and chloroform to each well of the cassette. For this operation, the hand is rotated to activate a paddle that is lowered into a well to stir the fermentation broth during addition of organic solvents. The robot then aspirates a several millilitre sample of the immiscible organic solvent and places it in a conical vessel. For each sample-transfer, a pipette manipulator on the hand picks up a pipette tip, uses it, and then discards it. The holder for the pipette tips is also an air manifold that directs heated air into the cups to evaporate the solvents. With the solvents evaporated, the hand is rotated to use the aerosolizing tip for the addition of reconstituting solvent. These reconstituted extracts are distributed to various bioassay laboratories.

### Integrated use of robotic systems in a high throughput approach towards drug discovery

*Mark Beggs, John Major, Colin Bath and Tina Hayden (High Throughput Screening Laboratory, Bioscience II, ICI Pharmaceuticals, Mereside, Alderley Park, Macclesfield, Cheshire SK10 4TG, UK)*

A range of robotic systems are being used to identify novel chemical leads against selected targets against which there are no obvious starting points for medicinal chemistry programs based programs.

Randomly submitted samples from the company's compound collection are solubilized in dimethyl sulfoxide using a Zymate II System. The use of appropriate barcoding technology permits the robot to solubilize samples of varying weights to give stock solutions of fixed concentrations. Working strength solutions are then automatically prepared from the stock solutions.

Assays are subsequently performed using a Tecan RSP5072 robot utilizing Amersham's Scintillation Proximity Assay Technology. Text compounds, controls and reagents are added to 6 × 16 well T-Trays which are sealed and counted in an LKB Pharmacia 1205 Betaplate scintillation counter. All data analysis is automatic and results are automatically uploaded onto a mainframe relational database.

Automation of these processes has resulted in a seven-fold increase in assay throughput as compared to manually operated screens. This throughput has been achieved without any further increase in staff.

### An automated method for the determination of a new antiepileptic drug candidate (CGP-33,101) in human plasma using high-performance liquid chromatography

*Linda A. Brunner (Development Department, Pharmaceuticals Division, Ciba-Geigy Corporation, Ardsley, NY, USA)*

An automated method utilizing laboratory robotics has been developed and validated for quantifying concentrations of a new antiepileptic drug candidate (CGP-33,101) in human plasma. The robotic system aliquots the biological sample, adds the internal standard (CGP-23,901), extracts the compounds from the basified biological matrix (pH 12) into an organic phase (dichloromethane: methyl t-butyl ether) and concentrates the extracts for reversed-phase, high performance liquid chromatography (HPLC) analysis. The laboratory robot is directly interfaced to the HPLC system, and the data are automatically collected and results calculated. Separation is achieved on a 3-um, Hypersil ODS (4·6 × 50 mm) column with ultraviolet (UV) detection of the drug and internal standard at 230 nm. Recovery and reproducibility assessments indicate good accuracy (overall mean relative recovery of 102·7%) and precision (coefficient of variation of 4·4 to 7·7%) over the CGP-33,101 concentration range of 50 to 4000 ng/ml, with a quantification limit of 50 ng/ml. The method has been successfully applied to a pharmacokinetic study in which normal volunteers received single, oral doses of 400–1200 mg CGP-33,101.

### An automated sample preparation and radiochemical HPLC analysis of Technetium $^{99m}$Tc-teboroxime

*R. Fisco, J. W. Sulner, D. Silowka, J. Troskosky, J. P. Zodda and M. N. Eakins (Bristol-Myers Squibb Pharmaceutical Research Institute Diagnostics Evaluation Department, New Brunswick, NJ 08903, USA)*

The sample preparation and radiochemical HPLC analysis of a new myocardial perfusion agent, $^{99m}$Tc-Teboroxime (CardioTec), has been automated using a shielded Zymark V Robotic System. Product specifications require $^{99m}$Tc-Teboroxime to be reconstituted with 1 ml of diluted eluate containing radiochemical concentration of 100 mCi $^{99m}$Tc-pertechnetate ($TcO_4$). The robot weighs a reaction vial, calculates the ratio of radioactive eluate and 0·9% saline contain 100mCi $^{99m}TcO_4$ in 1 ml, delivers the volume into the vial, and constantly adjusts the volume-ratio of eluate-to-saline for subsequent vials by incorporating the decay factor for $^{99m}$Technetium (T1/2 = 6 h) into each calculation. Following reconstitution, the robot weighs each reconstituted reaction vial to confirm the volume, vortexes the vials, heats the vials for 15 min at 100 °C in a specially constructed lead shielded block, cools them to room temperature and then sequentially delivers a 0·2 μl sample into each of two HPLC systems to determine the radiochemical purity of the sample. The reconstituted reaction vials are stored in a lead block for 6 h and then reassayed by sequentially delivering a 0·5 μl sample into each HPLC system.

The advantages of automating this procedure include: (1) decreasing the amount of radiation exposure to laboratory personnel; (2) relieving skilled staff from performing routine, tedious tasks; (3) increasing productivity by allowing unattended operation for extended periods of time; and (4) increased assay reproducibility by eliminating human error.

### Robotic sample preparation and HPLC analysis of Verlukast in human plasma

*Charles Lin, John Yeh-Kang Hsieh, Bogdan K. Matuszewski and Michael R. Dobrinska (Merck Sharp & Dohme Research Laboratories, Department of Drug Metabolism, West Point, PA 19486, USA)*

An automated sample preparation procedure was described for analysing plasma samples originating from metabolic disposition and bioavailability studies. A Zymate I Laboratory System was modified and upgraded to prepare plasma samples for the quantification of verlukast, a potent $LTD_4$ receptor antagonist.

The robot performed all necessary steps for the plasma protein precipitation and then transferred the supernatants to the Perkin-Elmer HPLC system by direct injection. Verlukast and internal standard were separated on a DyChrom Chemcosorb ODS-UH column (5 u, 4·6 mm × 150 mm) using a mobile phase consisting of methanol and ammonium phosphate buffer. Detection and quantification were carried out using a McPherson FL-750 Fluorometer and an HP 3357 Laboratory Automation System, respectively.

The system has been developed for unattended 24 h operation in the dark due to photosensitivity of verlukast. The overall accuracy and precision attained is 98·9 ± 0·2% for concentrations of 0·1 to 5 μg/ml. The analytical method has been demonstrated to have the sensitivity and specificity necessary to quantify plasma concentrations of the above compound following oral and I.V. administration of verlukast to normal subjects.

### Laboratory automation study

*Allan L. Greenberg (Laboratory Robotics Interest Group, NJ, USA) and Linda A. Brunner (New York-New England Lab Automation Interest Group, USA)*

Midwest Research Institute has conducted a survey on laboratory automation and its implementation. The survey was supported by both the Laboratory Robotics Interest Group of New Jersey and the New York-New England Lab Automation Interest Group.

The results from more than 2000 responses were presented. The information should provide users of laboratory automation technology with some valuable information and insights.

### Development of a customized Zymate II system to automate primary structure determination of recombinant proteins

*M. Reino, P. Diegelman, S. Pocchiari, M. J. Ehrke and J. W. Cowens (Grace Cancer Drug Center, Roswell Park Cancer Institute, Buffalo, NY 14263, USA)*

Quality control procedures for insuring the identity and purity of materials used as therapeutic agents are required by the FDA. The utilization of recombinant proteins in therapeutic applications is an area of rapid development; however, most of the procedures for the quality control of such materials require specially trained personnel and have not been automated. A Zymark Laboratory Automation System is being developed to automate the procedures used to determine the primary structure of proteins and polypeptides, i.e. amino acid analysis, enzymatic mapping, and sequencing. To accomplish this task, the automated system must successfully carry out a series of discrete chemical reactions for each analysis and since the size of sample available for analysis may be small, the PICOTAG system developed by Waters Chromatography Division has been used as the model for carrying out a series of chemical reactions on a microscale. In the PICOTAG system all reactions occur in a 6 × 50 mm Pyrex tube contained with a reaction chamber under inert atmosphere; reagents are removed after each reaction by a vacuum system. To adapt this approach to the Zymate II System, our laboratory collaborated with Zymark to modify standard stations to accommodate the chamber-tube assembly, to develop custom stations for vacuum evaporation and purging with inert gases, and to write the new software required for the robot to interact with the modified and customized stations. It has been demonstrated that the robotic system can carry out each step required for the amino acid analysis procedure. A synthetic 10-mer peptide has been analysed on the customized Zymate system and the results are completely comparable to those of the PICOTAG system. Studies are under way to determine the sensitivity and reproducibility of the analysis on the robotic system. Since the robotic system is capable of performing a series of modular operations (pipetting, weighing, mixing, heating, evaporating, etc.) in any order, the automation of enzymatic mapping and primary sequence determination are being developed on the same system.

### A robotic system for the automated DNA sequencing reactions

*S. Kajie and Y. Mizuno (Tonen Corporation, Saitama, Japan)*

The manual method for the DNA sequencing prior to the analysis by automated DNA sequencer is a dull and time-consuming procedure, and the process needs to be performed under dim environment for the light protection of fluorescent-labelled dye primer. Completion of the sequencing reactions is one of the rate-limiting steps in automated DNA sequencing.

To increase efficiency and reliability in automated DNA sequencing, the fluorescent-labelled DNA sequencing reactions have been automated including annealing reaction, elongation/termination reaction, and sample concentration by ethanol precipitation. The instrument is a Zymate II Laboratory Automation System modified to include handling of screw-capped microcentrifuge tube, high-speed refrigerated centrifuge, liquid sample processing, and thermal treatment. Reactions were performed in microtubes with screw cap, which prevented loss of liquid by evaporation and ensured mixing of microlitre quantities of solutions by means of Vortex and brief centrifugation. Sequencing samples were prepared by Sanger dideoxy method using *E. coli* DNA polymerase I Klenow fragment. The quality and reproducibility of the reactions were examined by an automated DNA sequencer, and sequence data of comparable quality to those of manual sequencing reaction was obtained.

### Use of robotics for the automation of the angiotensin II receptor binding assay

*Mary Jo Wildey, John Nygaard, Deborah Steinberg, Michael Greenstein and William M. Maiese (Lederle Laboratories, Middletown Road, Pearl River, NY 10965, USA)*

In the pharmaceutical industry, high-throughput screening is a key step in the discovery of novel agents with therapeutic utility. Many detection systems, such as receptor binding assays, involve repetitive procedures which can be readily conducted by laboratory robotics to achieve the desired throughput goals. An automated protocol for the [125](sar$^1$, ile$^8$)Angiotensin II receptor binding assay, developed by Lederle's Cardiovascular Research Section, is currently being run. A Beckman Biomek SL robot is employed to accurately pipette test samples, ligand, and receptor to the wells of a microtitre plate. After a timed incubation, all 96 wells of the assay plate are simultaneously harvested onto a filtermat and washed by a Tomtec 96 well harvester. Receptor-bound ligand retained on the filtermats is subsequently quantitated by scintillation counting on a LKB Betaplate reader. Data is automatically transferred to a database on Lederle's VAX mainframe for subsequent analysis, permanent storage, and report generation. For all automation projects, criteria for acceptability, which require the robot to meet or exceed the statistical limits for variability established for the manual version of each assay, have been devised. In an initial assessment of this system, a random set of synthetic compounds were evaluated at the rate of 350 samples per hour. Active candidates were further analysed robotically to determine their $IC_{50}$'s. Assays relevant to all therapeutic areas within Cyanamid are under consideration for automation and subsequent testing of synthetic compounds and natural products.

### Robotics and automation of ligand binding assays

*Randy A. Turner and Robin A. Felder (University of Virginia, Charlottesville, VA, USA)*

Ligand binding has become an indispensable tool in basic research laboratories and the pharmaceutical industry. Assays measuring the binding of fluorescent or radioisotope labelled ligands to cell surface receptors allow sensitive and rapid screening of drug potency and specificity. Robots and the peripheral devices with which they interact can be used to automate various aspects of receptor assays. Dopamine receptors were studied in rat brain and kidney using a variety of dopamine agonists and antagonists in competition with $^{125}$I-labelled Sch 23982. Identical assays were performed manually and with a Hamilton Microlab AT pipetting robot. The Microlab AT was allowed to make serial drug dilutions, including tip wiping, tip changing, and sample mixing. It was also allowed to dispense samples and reagents onto microtitre plates. All other steps in the assays were performed manually. For a twelve drug assay, the robot was more than six times faster diluting drugs than a human technician, and was more than eight times faster than a human in dispensing 50 ul aliquots of drug, tissue, competing ligand, and buffer into each well of a standard 96-well microtitre plate. The competition curves generated by both manual and machine assays were in good agreement; however, the repetition standard deviations were significantly lower for the Microlab AT (manual = 513 ± 84 CPM, machine = 485 ± 20 CPM, $p \leq 0{\cdot}05$, $N$ = 3). Use of the robot allowed smoother curves to be generated with fewer repetitions ($N$ = 2 vs. $N \geq 4$) and smaller volumes (15 ul vs. 50 ul). Scatchard plots from the robot data yielded dissociation constants and maximum receptor densities in agreement with literature values. Precision and accuracy tests yielded coefficients of variation of 9% to less than 0·5% for 5 microlitre volumes, depending upon the pipetting protocol; and significantly better values were obtained for larger volumes. Other aspects of radioligand binding automation, including the use of cell harvesters and microtitre based scintillation and radioactivity counters, were also discussed.

### Handheld computer RS232C interfacing to an analytical balance

*Albert Marcus Morrishow and Wah Sang Lee (Mount Sinai School of Medicine, New Yok, NY, USA)*

A Hewlett-Packard Company (HP) HP48SX handheld computer workstation was interfaced to a Sartorius R200D semi-micro analytical balance via RS232C. The handheld computer functions as the front-panel (control keys and display) for the analytical balance. Handheld computer commands, to tare and send ASCII text from the analytical balance, are embedded within application specific user programs. Application tasks are invoked via program softkeys. Analytical balance acquired sample weights, are sent to the handheld computer, where the received text, is simply stored, or is preprocessed and then stored. With software available from HP, data can be sent from the handheld computer to IBM and IBM-compatible PCs and Apple Macintosh Computer workstations.

### Handheld computer RS232 interfacing to a gas chromatograph mass spectrometer (GC/MS) workstation

*Albert Marcus Morrishow and Wah Sang Lee (Mount Sinai School of Medicine, New York, NY, USA)*

A Hewlett-Packard Company (HP) HP48SX handheld computer was interfaced to a HP59970B Gas Chromatograph/Mass Spectrometer (GC/MS) Chemstation (workstation) via RS232. ASCII test of integrated GC/MS acquired data are sent from the GC/MS Chemstation to the HP48SX, where the received text is simply stored, or is preprocessed and then stored. With software available from HP, data can be sent from the HP48SX to IBM and IBM-compatible PCs and Apple Macintosh Computer workstations.

### The use of a Zymark II robot to perform automatic titration of gastric juice samples

*Mark Hamilton, Antonio Guglietta, Kim Collins, Mary Gerrelts, Lynne Hupe, Jim Fergus and Jim Marks (Warner-Lambert/Parke-Davis, Pharmaceutical Research Division, 2800 Plymouth Road, Ann Arbor, MI 48106-1047, USA)*

Gastric secretory studies are widely used in research laboratories to obtain data on gastrointestinal physiology and pharmacology. Determination of hydrogen ion concentration and gastric acid output requires base titration of an aliquot of gastric juice to pH 7·0. This procedure, when done manually, is very time consuming and only a limited number of samples can be handled at any given time.

This task has been automated utilizing a Zymate II robot outfitted with a remote computer interface. The entire process is controlled by a master program that computes the amount of NaOH to add based on output from a pH meter. A comparison of results of titrations performed manually and by the robot were almost identical.

### Automated sample preparation for colorant analysis with BenchMate workstation

*Dean B. Higgins and Kathryn L. Jansen (Manufacturing Quality Assurance Organization, Eastman Kodak Company, Rochester, NY, USA)*

A Zymark BenchMate is being used to prepare coloured electrographic toner samples for percentage colorant determination by spectrophotometry. The coloured toner samples must be dissolved in either N,N-dimethylacetamide or concentrated sulfuric acid for this analysis. Use of the BenchMate for sample preparation minimizes analyst contact by adding and mixing the solvents automatically. Also, the total volume of solvent used (and waste to be disposed of) can be reduced by a factor of five, from 100 ml to 20 ml per sample. Using BenchMate preparation, no bias is observed and initial experiments indicate reduced variability as compared to manual sample preparation. Optimization of the method and analytical crossover results were described.

### Use of Zymark BenchMate with Hewlett-Packard diode array spectrophotometer for routine UV-VIS-NIR measurements

*J. C. Hammontree and C. G. Zimba (Polaroid Corporation, Chemical Research Division, 750 Main Street 3D, Cambridge, MA 02139, USA)*

Absorption spectra of powders dissolved in various organic solvents are required in the authors' laboratory. Using manual methods to prepare and measure the solutions, it typically takes 30–60 min for each sample. At present, approximately three-quarters of a man-year is spent doing these analyses with the vast majority, 80–90%, of this time consumed in the preparation of solutions. The Zymark BenchMate allows the solutions to be prepared under automated control and then delivered, via a flow cell, to the spectrophotometer. By reducing the time required to prepare solutions to 10% of the present commitment, the BenchMate will allow two associate scientists now committed to this work to be redirected to projects of a more appropriate skill level. Additionally, by allowing unattended overnight data acquisitions, the BenchMate allows more rapid response for the routine service work that our laboratory performs. Furthermore, sample quantity requirements are reduced, lowering the amount of analyte required for the analysis and lowering the amount of waste organic generated. Progress toward integrating the Zymark BenchMate to a Hewlett-Packard diode-array spectrophotometer for routine UV-VIS-NIR absorption measurements were discussed.

### Capacitive proximity sensor aids robot navigation

*Bill Mordan and John Shigeura (Applied Biosystems, Foster City, CA, USA)*

Fluid-handling laboratory robots require positional information regarding the liquid volumes that they work on, as well as various solid objects such as trays and tubes. This poster described how a capacitive proximity sensor provides this feedback in a robotic instrument that executes DNA sequencing reactions. The software data structures that use the feedback are illustrated.

Operating through a stainless steel pipette tip manipulated by the robot, the capacitive sensor enables the robot to locate the surface of each fluid volume in its workspace. This feedback reduces tip contamination and alerts the instrument controller when pipetting failures have occurred. The sensor also allows the robot to locate datum planes on the worksurface and on each well tray. With this information, the instrument controller can compensate for errors in the tray and tip positions, thereby improving the accuracy of robot motions in the wells. Lastly, the sensor is used to identify well trays by detecting the coded profile of each tray.

### Automated assay for Vigabatrin and MDL 17,637 in Sabril tablets using the Zymark BenchMate

*W. Al Kentrup and Jeff Huth (Analytical Development, Marion Merrell-Dow, 2110 E. Galbraith Road, Cincinnati, OH 45215, USA)*

An automated method has been developed on the Zymark BenchMate for the release/stability and content uniformity assay of vigabatrin and its degradation product MDL 17,637 in sabril tablets. The BenchMate samples an aliquot of the solubilized sabril tablet, filters the solution, performs the appropriate dilutions, and injects an aliquot of the processed sample into the chromatograph. A Whatman Partsil-10 SCX column is used to facilitate the separation of vigabatrin, MDL 17,637, and excipient materials with UV detection at 210 nm. The sample processing time is approximately 10 min, and is equal to the chromatographic run time. As a result, instrument lag-time is minimized.

Using the developed method, linearity studies for both vigabatrin and MDL 17,637 produced correlation coefficients of 1·000. Precision of the method was determined both chromatographically as well as using the gravimetric function of the weigh station of the BenchMate. When ten aliquots of the same sample solution were processed by the BenchMate, peak area counts for vigabatrin and MDL 17,637 had relative standard deviations of 0·25% and 0·41% respectively. In six separate analytical runs ($N$ = 7–50 samples), 0·9 ml of solution sampled for dilution ('conc asp') produced an average relative standard deviation of 0·27%. Similarly, the 2·25 ml of diluted solution dispensed ('sln disp') produced an average relative deviation of 0·26%. Recovery data for vigabatrin from spiked placebo samples produced an overall recovery of 100·4% ($N = 30$). For MDL 17,637, the average recovery from spiked placebo samples ($N = 30$) over the range from 0·03% to 2·64% (w/w) MDL 17,637/active drug was 97·4%. For the higher concentration range of 0·11% to 11·90% (w/w) an average recovery of 98·8% was obtained ($N = 6$).

The developed method has proven to be linear, accurate and precise. In addition, a cost reduction in labour and reagents has been realized using the automated method over the conventional manual method.

### Analysis of urinary catecholamines using Varian's AccuCAT Column for sample preparation with Zymark's BenchMate workstation

*Rachel A. Grace (Varian Sample Preparation Products Harbor City, CA, USA) and Lynn Jordan (Zymark Corporation, Hopkinton, MA 01748, USA)*

Catecholamines are neurotransmitters used by the body to regulate motor response and metabolism. Overproduction of catecholamines can cause hypertension or congestive heart failure, and can also be indicative of the adrenal gland tumour known as pheochromocytoma. Quantitation of catecholamines in urine is used as an aid in diagnosis.

Catecholamines in the body are converted to an acidic or basic metabolite by catechol-O-methylation. In the past, multiple solid phase extraction (SPE) technologies were necessary to quantitate 'total catecholamines'. Newer methodologies use the concept of multimodal solid phase extraction columns. The AccuCAT column is a mixed mode bed column for isolation of catecholamines, and allows one SPE cartridge to be used for determination of catecholamine levels.

With today's trends towards automation, and the increasing concerns about handling bodily fluids, the sample preparation and HPLC injection of catecholamines was automated with the BenchMate Workstation.

The patient samples were diluted and pH adjusted prior to placement on the BenchMate Workstation. The BenchMate Workstation conditioned the AccuCAT columns, performed the SPE, diluted the eluant and injected the sample onto the HPLC.

This paper described the combination of two technologies: the use of the AccuCAT columns and the BenchMate Workstation for automation of the Catecholamine Analysis. The paper focused on the determination of Vanillylmandelic Acid, the most common acidic metabolite of catecholamines. The poster demonstrated the transfer of the manual method to the BenchMate Workstation. The automated method achieves good linearity and high percentage recoveries.

### Aflatoxin determination using Vicam's Aflatest-P immunoaffinity columns and Zymark's BenchMate workstation

*Lynn Jordan (Zymark Corporation, Hopkinton, MA 01748, USA) and Kevin F. Donahue (Vicam L.P., Somerville, MA 02145, USA)*

Aflatoxins are naturally occurring carcinogens produced by moulds found in grains, nuts and other commodities. Monitoring aflatoxin contamination is essential for production of quality food products and for compliance with FDA guidelines.

This poster demonstrated the combination of two technologies for improved aflatoxin analysis: Aflatest immunoaffinity chromatography for aflatoxins and the BenchMate Workstation for automated sample processing. The result is increased sample throughput and highly reproducible data. Aflatest immunoaffinity columns are highly selective to aflatoxin and permit rapid and efficient sample clean up. The affinity column clean up method does not require the use of hazardous chemicals or lengthy sample preparation steps of some of the more traditional aflatoxin methods. Aflatest columns utilize monoclonal antibodies with a high specificity for aflatoxins B1, B2, G1 and G2. This method has received official first action approval by the AOAC.

Combining the BenchMate Workstation with the Aflatest columns offer a number of additional benefits. With the BenchMate Workstation's ability to precisely control flow rates used to load, wash and elute columns, studies were easily set up to optimize the method. An additional feature of the automated method is the ability of the workstation to make direct HPLC injections, eliminating another sample handling step. Use of the BenchMate Workstation to automate the aflatoxin analysis can mean a time saving of 4 h a day with 30 analyses per day.

Data was presented from studies used to determine the optimum loading flow rate for the Aflatest columns. Once an optimized method was determined, linearity studies and a comparison of the manual method to the BenchMate method was done. With the BenchMate automated method, the data shows an improvement in the reproducibility, along with a time saving for the analyst. The consistency of the results achieved can lead to high levels of confidence in producing a quality food product.

### Automation of the assay for the determination of enzyme activity of modified superoxide dismutase injectable drug product by the inhibition of cytochrome C reduction method

*Charles Houck, Martin Echols, Timothy Dankanich and William Pickens (Sterling Research Group, Malvern, PA, USA)*

A robotic system has been developed which can conduct unattended, the determination of enzyme activity of polyethylene glycol superoxide dismutase (PEG-SOD) in an injectable drug product. Modified superoxide dismutase is currently being investigated as a potential anti-inflammatory drug and antioxidant for reperfusion of ischaemic tissue.

The assay is based upon the method developed by McCord and Fridovich. In this method, xanthine oxidase acting aerobically upon xanthine generates superoxide radicals, this reaction can be followed at 550 nm by monitoring the reduction of cytochrome c. PEG-SOD, by competing for the superoxide radicals, will inhibit the reduction of cytochrome c. The system produces high accuracy and precision both interday and intraday.

A Sun 386i workstation controls a Hewlett-Packard Widmark robot, collects time based data from a Perkin-Elmer Lambda 2 spectrophotometer, processes the data, analyses the results and then makes appropriate decisions concerning the assay conditions based upon those results. This poster described the software developed, the hardware and how the Sun 386i workstation is being used as a test station controller to optimize the reaction conditions and ensure the quality of the results generated.

### Preparation of tablet samples for HPLC analysis using a Zymate PySystem in a custom mode and supplemented with a BenchMate workstation

*B. A. Brown, J. E. Curley and L. J. Kostek (Pfizer, Inc., Groton, CT, USA)*

The Pfizer Analytical Research & Development Robotic's Laboratory has developed a diversified path to automation of tablet sample preparation. Two automated methods have been developed for sample preparation using a PyTechnology for totally automated testing or a Zymark BenchMate for semi-automated preparations. These systems can be used separately or together depending on the needs of the product.

A PySystem has been configured to perform a totally automated sample prep procedure. Custom programs have been written and integrated with some of the Zymark Pysoftware to perform all the necessary steps of sample preparation. This system will weigh a tablet and add the dissolving solvent. The mixture is shaken and a portion of the extract is centrifuged. The clarified solution is diluted and vortexed. The final solution is capped and stored for HPLC end analysis. The Zymark BenchMate may be incorporated into this procedure if centrifugation proves to be inadequate and additional filtration is necessary. The BenchMate has the capability of performing filtration and dilution of a previously dissolved solution, which has been prepared either manually or by the PyTechnology sample prep system.

This poster presented a detailed description of both the Zymate sample prep system and the BenchMate procedures. Validation experiments along with supporting data were provided to indicate system suitability. These systems are an alternative to manual preparation of samples for HPLC analysis.

### Analysis of climate gases $CO_2$, $N_2O$ and $CH_4$ by wide bore capillary gas chromatography

*Bishal K. Sitaula, Luo Jiafa and Lars Bakken (Agricultural University of Norway, 1432 Aas-NLH, Norway)*

A gas chromatograph method is described, which gives sensitive and rapid (3·3 min) measurement of the three greenhouse gases $CO_2$, $CH_4$ and $N_2O$. A wide bore capillary column operated at optimal conditions gave a sufficient separation to allow a switch of the column flow between two detector systems (TCD and FID in series, and ECD) during the chromatogram, thus all three gases could be analysed with a single sample injection. Application of a peristaltic pump allowed gas samples to be transferred directly from gas sampling vials, through a drying agent ($MgClO_3$) and into the injection loop (0·2 ml). Back flushing of the injection system (pump and drying agent) with He between each injection ensured minimal carry over between samples (<0·6%). Experience with storage of gas samples in vials with butyl rubber septa was described.

### Payback and benefits of a robotic dissolution system

*Ronnie McDowell (Sandoz Pharmaceuticals, QA/Methods Development, East Hanover, NJ, USA)*

As part of the justification for the purchase of a robotic dissolution system it was determined that such a system would result in a net gain in productivity of 1·5 to 1·75 analyst per year. Based upon this assumption, it was further determined that full payback of such a system could be attained in less than two years. This poster presented the authors' experience with the system after one year and included the initial installation, calibration and validation of the system. Also presented were the results of a typical month of automated dissolution testing in our quality assurance laboratory. This data was used to determine whether the forecasted payback period is attainable.

### Automated dissolution testing using USP method I (baskets) and real-time HPLC analysis

*D. W. Barrow and W. S. Conder (Bristol-Myers Squibb, P.O. Box 191, New Brunswick, NJ 08903-0191, USA)*

A fully automated *in vitro* dissolution test for sustained-release capsules of various potencies has been achieved using a Zymark Zymate I Plus robot (Zymark Corporation, Hopkinton, MA) and on-line HPLC analysis. The test is performed in 1000 ml of dissolution media using the USP XXII (basket) method I at 50 rpm and at 37·0 ± 0·5 °C. Telescoping basket shafts are utilized to initiate sample testing at specific time intervals.

The system is capable of performing four complete dissolution tests in 24 h without operator intervention. This represents a 200% increase in productivity over conventional manual test methods.

### Making robots smarter: a critical analysis of the force feedback signals from the Zymate II robot and their use in error detection and correction

*Naved A. Surve and Dennis S. France (Department of Lipids and Lipoprotein Metabolism, Sandoz Research Institute, East Hanover, NJ 07936, USA)*

Laboratory robots are being adapted for increasingly complex uses that requrie extreme precision. While these adaptations might be crucial to the success of an assay, they also increase the chances of failure for the robot. If robots were able to foresee conditions where they were about to crash, newer and more complex assays would become reliable. This would open the door for the robotization of more assays, freeing the time of laboratory workers for more inventive tasks.

The Zymate II System provides feedback data on forces exerted by the arm. By knowing what forces are exerted under normal conditions and conditions of stress, it is possible to create error checking programs which effectively give the robot the ability to recognize and correct errors.

Both arm and hand force feedback parameters on two microplate management systems have been quantitatively mapped. The limits, precision, reproducibility, and utility of these parameters in the context of robotic microplate management were presented. In addition, specific examples of error detection and correction were discussed. A preliminary comparison was made between the force feedback potentials of the Zymate II arm and the faster Zymate XP arm.

### Macintosh control of the Zymate robot: construction of a multiple peptide synthesizer

*Ronald N. Zuckermann, Michael A. Siani, Steven C. Banville and Janice M. Kerr (Chiron Corporation, 4560 Horton Street, Emeryville, CA 94608, USA)*

A multiple peptide synthesizer has been constructed by integrating a Zymate robot arm and a peptide synthesis station under the control of a Macintosh computer. The Macintosh software co-ordinates the movements of the Zymark robot arm (via a remote computer interface with the Zymark controller), the switching of over 40 solenoid valves and the monitoring of sensors (via an internal digital I/O board). The Zymark hands are used to deliver solvent from pressurized spigot lines and to pipette amino acid solutions from reservoirs to an array of reaction vessels. Liquid dispensing, reagent mixing and solvent removal are controlled from the Macintosh digital I/O board. The Macintosh was chosen because it permits a friendly user-interface and supports powerful programming languages. C language has been used to conveniently accommodate peptide sequence, position and quantity information in multi-dimensional arrays which are not supported by the EasyLab environment.

### Using a Macintosh IIx to perform off-line remote programming

*J. R. Ormand, A. E. Borgwardt, B. E. Kropscott and P. L. Morabito (The Dow Chemical Company, Health and Environmental Sciences, Midland, MI 48674, USA)*

A Macintosh IIx computer (Apple Computer, Inc., Cupertino, CA), has been used in successful off-line remote programming of a Zymate II System (Zymark Corporation, Hopkinton, MA). Several applications have been edited using SoftPC EGA/AT v1.4 emulating software (Insignia Solutions, Inc., Sunnyvale, CA).

When Zymark introduced the System V controller, it provided an interface between the robotic controller and a common computer environment, the IBM PC or PC-compatible computer. This interface offered the advantage of having applications on the PC available to the user that could not be offered by the Zymate controllers. Applications such as word processing, and data manipulation software can now be used in conjunction with the System V software to enhance the user's capabilities. Another advantage of a PC-based system is the convenience of programming from a remote location and then transferring the programs to the robotic system.

To accomplish remote editing on a Macintosh, the System V software was loaded onto the PC emulator hard drive. From the file conversion utility in the System V menu, the Zymate II dictionary (on a 5·25 in diskette) was converted to a binary .ZYD dictionary using an Apple 5·25 in PC disk drive. The newly created .ZYD dictionary was then opened and edited by the System V software on the Mac. To enhance dictionary editing, Microsoft Ward v4.0 (Microsoft Corporation, Redmond, WA) was used in conjunction with the System V software to create and edit program text which was cut, copied, and pasted to and from the System V editor using the Macintosh's clipboard.

To take this a step further, Zymark file conversion programs were used to convert the binary .ZYD dictionary to an editable text file (.ZYE dictionary) and vice versa. By opening the .ZYE text file in Microsoft Word, program text, positional coordinates, rack definitions (indexes and coordinates), and module commands were edited on the Macintosh as a word processing document. Following editing, the .ZYE file was converted back to the .ZYD dictionary and finally back to the Zymate II 5·25 in disk.

An advantage to the dictionary conversion method of editing is the ability to edit the entire dictionary as one document. This approach allows the user to edit in a powerful word processing environment instead of editing in the System V editor. Another advantage to dictionary conversion is the ability to transfer positional code and rack definitions between dictionaries, which cannot be accomplished with any of Zymark's editors. The transfer of programs also allows the user to create PyAppendable disks for custom applications. A disadvantage of editing a .ZYE file is that the format is more difficult to manage than the format in the System V editor. Also, when editing the .ZYE file in a word processing application, care must be taken to insure that the application does not delete or add control characters to the file. Another disadvantage to this method of editing is that the interaction with the System V software through the PC emulator is slow.

This poster described the methods used, as well as the advantages and disadvantages of off-line remote programming on the Macintosh IIx.

